# Oncogenic CARMA1 couples NF-κB and β-catenin signaling in diffuse large B-cell lymphomas

**DOI:** 10.1038/onc.2015.493

**Published:** 2016-01-18

**Authors:** M K Bognar, M Vincendeau, T Erdmann, T Seeholzer, M Grau, J R Linnemann, J Ruland, C H Scheel, P Lenz, G Ott, G Lenz, S M Hauck, D Krappmann

**Affiliations:** 1 Research Unit Cellular Signal Integration, Institute of Molecular Toxicology and Pharmacology, Helmholtz Zentrum München—German Research Center for Environmental Health, Neuherberg, Germany; 2 Translational Oncology, Department of Medicine A, Albert-Schweitzer Campus 1, University Hospital Münster, Münster, Germany; 3 Cluster of Excellence EXC 1003, Cells in Motion, Münster, Germany; 4 Department of Physics, Philipps-University Marburg, Marburg, Germany; 5 Institute of Stem Cell Research, Helmholtz Zentrum München—German Research Center for Environmental Health, Neuherberg, Germany; 6 Institut für Klinische Chemie und Pathobiochemie, Klinikum rechts der Isar, Technische Universität München, Munich, Germany; 7 German Cancer Consortium (DKTK), Heidelberg, Germany; German Center for Infection Research (DZIF), partner site Munich, München, Germany; 8 Department of Clinical Pathology, Robert-Bosch-Krankenhaus, Dr Margarete Fischer-Bosch Institute of Clinical Pharmacology, Stuttgart, Germany; 9 Research Unit Protein Science, Helmholtz Zentrum München—German Research Center for Environmental Health, Neuherberg, Germany

## Abstract

Constitutive activation of the antiapoptotic nuclear factor-κB (NF-κB) signaling pathway is a hallmark of the activated B-cell-like (ABC) subtype of diffuse large B-cell lymphomas (DLBCL). Recurrent oncogenic mutations are found in the scaffold protein CARMA1 (CARD11) that connects B-cell receptor (BCR) signaling to the canonical NF-κB pathway. We asked how far additional downstream processes are activated and contribute to the oncogenic potential of DLBCL-derived CARMA1 mutants. To this end, we expressed oncogenic CARMA1 in the NF-κB negative DLBCL lymphoma cell line BJAB. By a proteomic approach we identified recruitment of β-catenin and its destruction complex consisting of APC, AXIN1, CK1α and GSK3β to oncogenic CARMA1. Recruitment of the β-catenin destruction complex was independent of CARMA1-BCL10-MALT1 complex formation or constitutive NF-κB activation and promoted the stabilization of β-catenin. The β-catenin destruction complex was also recruited to CARMA1 in ABC DLBCL cell lines, which coincided with elevated β-catenin expression. In line, β-catenin was frequently detected in non-GCB DLBCL biopsies that rely on chronic BCR signaling. Increased β-catenin amounts alone were not sufficient to induce classical WNT target gene signatures, but could augment TCF/LEF-dependent transcriptional activation in response to WNT signaling. In conjunction with NF-κB, β-catenin enhanced expression of immunosuppressive interleukin-10 and suppressed antitumoral CCL3, indicating that β-catenin can induce a favorable tumor microenvironment. Thus, parallel activation of NF-κB and β-catenin signaling by gain-of-function mutations in CARMA1 augments WNT stimulation and is required for regulating the expression of distinct NF-κB target genes to trigger cell-intrinsic and extrinsic processes that promote DLBCL lymphomagenesis.

## Introduction

Constitutive activation of the nuclear factor-κB (NF-κB) pathway is a hallmark of different lymphoma subtypes. Diffuse large B-cell lymphomas (DLBCL) account for the largest number of non-Hodgkin lymphomas, which were classified into two major sub-entities: the activated B-cell-like (ABC) and the germinal center B-cell-like (GCB) DLBCL.^1^ Whereas most GCB DLBCL do not rely on NF-κB signaling, survival of ABC DLBCL is highly dependent on constitutive NF-κB activation.^2^ Canonical IκB kinase/NF-κB signaling in ABC DLBCL cells is often triggered by chronic B-cell receptor (BCR) signaling pathway.^3^ Accordingly, BCR-signaling components like CD79A/B, SYK (spleen tyrosine kinase), BTK (Bruton's tyrosine kinase) and PKCβ (Protein kinase C β) are indispensable for survival of ABC DLBCL cells.^3, 4, 5^ BCR signaling promotes permanent activation of the CARMA1-BCL10-MALT1 (CBM) complex that bridges upstream signaling events to the IκB kinase complex.^4^

The key role of constitutive NF-κB activation in ABC DLBCL cells is confirmed by recurrent somatic mutations.^6^ Activating upstream mutations have been detected in the BCR adaptors CD79A and CD79B (~21% of ABC cases) or the innate immune adaptor MYD88 (~30% ABC cases).^3, 7^ Also, inactivating mutations in the tumor suppressor A20, a negative regulator of NF-κB signaling, have been found in ABC DLBCL.^8^ About 10% of ABC DLBCL and ~4% of GCB DLBCL patients carry gain-of-function mutations in the scaffold protein CARMA1/CARD11.^9, 10^ Under physiological conditions, CARMA1 undergoes a phosphorylation-induced conformational change to recruit BCL10-MALT1 upon antigen stimulation in B and T cells.^11^ Oncogenic mutations are all localized within the coiled-coil (CC) domain of CARMA1 and are acting presumably by changing the conformation of the CARMA1 scaffold to allow stimulus-independent recruitment of BCL10-MALT1 and thus permanent CBM assembly.^9, 12^ Furthermore, CARMA1 mutations render ABC DLBCL cells resistant to inhibition of upstream kinases like SYK, BTK or PKCβ.^3, 13, 14^ Thus, quite in contrast to CD79A/B mutations, growth of CARMA1 mutated ABC DLBCL does no longer rely on a functional BCR, which underscores the potency of this oncogene.^3^

As CC mutations are thought to affect the scaffolding function of CARMA1, we took a mass spectrometry approach to search for novel interaction partners of active CARMA1 in BJAB cell system that provide an *in vitro* model system to analyze the function of oncogenes.^15^ We found a robust recruitment of the β-catenin destruction complex and stabilization of β-catenin in oncogenic CARMA1-transduced BJAB as well as in ABC DLBCL cell lines. In most cells, β-catenin is constantly degraded in the cytoplasm, but β-catenin stabilization upon WNT signaling promotes its function as co-activator of TCF/LEF transcription in the nucleus.^16^ Deregulations in WNT signaling and enhanced β-catenin amounts are found in many human cancers including hematologic malignancies.^17, 18^ We show here that stabilization of β-catenin by oncogenic CARMA1 engages a novel cross-talk between NF-κB and WNT pathways in DLBCL that can contribute to ABC DLBCL lymphomagenesis.

## Results

### Oncogenic CARMA1 recruits the β-catenin destruction complex and stabilizes β-catenin

**Figure 1:**
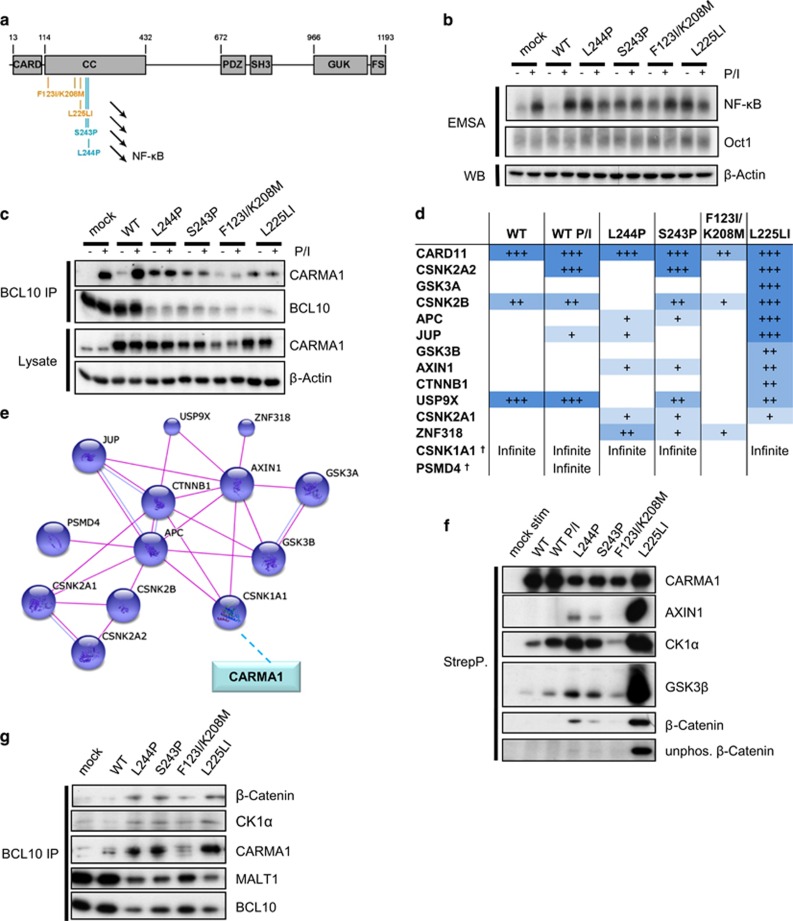
Proteomic analysis defines a functional interaction of oncogenic CARMA1 with the β-catenin destruction complex. (**a**) Schematic depiction of CARMA1 domains and generated ABC (blue) or GCB (orange) DLBCL-derived mutations of the coiled-coil (CC) domain. CARD: caspase recruitment domain; SH3: SRC homology 3; GUK: guanylate kinase; FS: Flag-Strep-Tag. (**b**) Oncogenic CARMA1 promotes constitutive NF-κB activity. NF-κB DNA binding was assessed in EMSA of unstimulated or 60 min P/I stimulated transduced BJAB cells. Oct1 EMSA was performed for control. (**c**) Oncogenic CARMA1 is constitutively recruited to BCL10-MALT1. CBM complex formation was assessed in stimulated (15 min P/I) or unstimulated transduced BJAB cells and analyzed in western blot after BCL10 IP. (**d**) LC-MS/MS identifies CARMA1 interaction partners consisting of proteins surrounding the β-catenin destruction complex. Three independent Strep-precipitation experiments were conducted in CARMA1 WT, WT stimulated (30 min P/I) and four different oncogenic CARMA1 mutant-transduced BJAB. The ratio of mean peptide abundances of each sample related to the mock precipitation control was calculated and depicted with +=2–10, ++=10–50 and +++=>50. Infinite stands for incalculable values, because no peptides were detected in mock control. † marks proteins that were identified by one single peptide. (**e**) Interaction database analysis of identified CARMA1 interaction partners emerges a network of proteins surrounding the β-catenin destruction complex. Known and predicted protein interactions using string database (string-db.org) was calculated based on experimental data with a medium confidence score. Only significant interactions (*P*<0.05) were used for string analyses. Published CK1α-CARMA1 interaction was integrated manually with dashed line. (**f**) Oncogenic CARMA1 interacts with proteins of the β-catenin destruction complex. CARMA1 StrepTactin pull-down in transduced BJAB cells and associated proteins were analyzed in western blot. (**g**) Proteins of the β-catenin destruction complex are associated to the CBM complex. The interaction of CK1α and β-catenin to the CBM complex was assessed in transduced BJAB cells and analyzed in western blot after BCL10 IP.

To identify oncogenic mechanisms of CARMA1-activating mutations, we cloned a panel of DLBCL patient-derived mutations that all affected the CC domain of the CARMA1 scaffold (Figure 1a).^9^ Two ABC-derived (L244P and S243P) and two GCB-derived (F123I/K208M and L225LI) CARMA1 mutants were expressed in the GCB DLBCL cell line BJAB that lacks chronic BCR signaling and constitutive NF-κB activation. Lentiviral transduction led to consistent infection rates of >95% as determined by fluorescence-activated cell sorting staining of the co-expressed human ΔCD2 surface marker (Supplementary Figure S1). CARMA1 WT and empty vector (mock) served as controls and all proteins were fused to a C-terminal FLAG-StrepTagII (FS) epitope for detection and affinity purification.

CARMA1 WT and mutants were expressed in BJAB cells at equivalent levels as judged by western blotting after harsh cell lysis (Supplementary Figure S2a). Cell lysis under less-stringent co-immunoprecipitation (co-IP) conditions reduced amounts of the CARMA1 F123I/K208M mutant owing to an enhanced recruitment to insoluble aggregates as observed by confocal immunofluorescence microscopy (Supplementary Figures S2b and c). Despite a strong overexpression of all CARMA1 proteins, CARMA1 WT was unable to drive constitutive CBM formation and NF-κB activation, but cells were still responsive to PMA/ionomycin (P/I) stimulation (Figures 1b and c). Only oncogenic CARMA1 mutants promoted constitutive NF-κB activation and BCL10 binding that were not further enhanced by P/I stimulation (Figures 1b and c).

We purified cellular CARMA1 complexes from transduced BJAB cells by StrepTactin pull-down and identified interaction partners by mass spectrometry using CARMA1 WT (± P/I) and the four oncogenic CARMA1 mutants. Three independent experiments were performed yielding a list of 33 CARMA1 interaction partners that were found consistently and significantly in three independent experiments to associate with at least one CARMA1 construct (Supplementary Table S1). Although CARMA1-BCL10 interaction was readily identified after BCL10 co-IP (Figure 1c), chronic activation of oncogenic CARMA1 induced BCL10 degradation,^19, 20^ which hampered a reliable BCL10 detection in mass spectrometry. Most interaction partners were identified in association with CARMA1 L225LI and many interactors were confirmed in the CARMA1 mutants L244P, S243P and F123I/K208M (Supplementary Table S1 and Supplementary Figure S3a).

Pathway analyses revealed one major interaction map that strongly associated with CARMA1 L225LI and more weakly to all other CARMA1 mutants consisting of proteins functionally linked with WNT/β-catenin network (Figures 1d and e). The interaction included β-catenin (CTNNB1) and main components of the β-catenin destruction complex, namely APC, AXIN1, GSK3α/β, CK1α as well as other regulators of WNT signaling. By StrepTactin pull-down and western blotting we verified the interaction of AXIN1, CK1α, GSK3β and β-catenin with CARMA1 (Figure 1f). Even though binding of the β-catenin destruction complex was predominantly obtained with CARMA1 L225LI, interaction was also seen with other oncogenic CARMA1 mutants or CARMA1 WT after P/I stimulation. The reduced co-precipitation especially of CARMA1 F123I/K208M was due to the stronger accumulation of these mutants in aggregates that were less-soluble under co-IP conditions (see Supplementary Figures S2a and b). Using anti-BCL10 IP, we confirmed that CK1α and β-catenin are associating with all four oncogenic CARMA1 mutants in the context of the CBM complex (Figure 1g).

**Figure 2:**
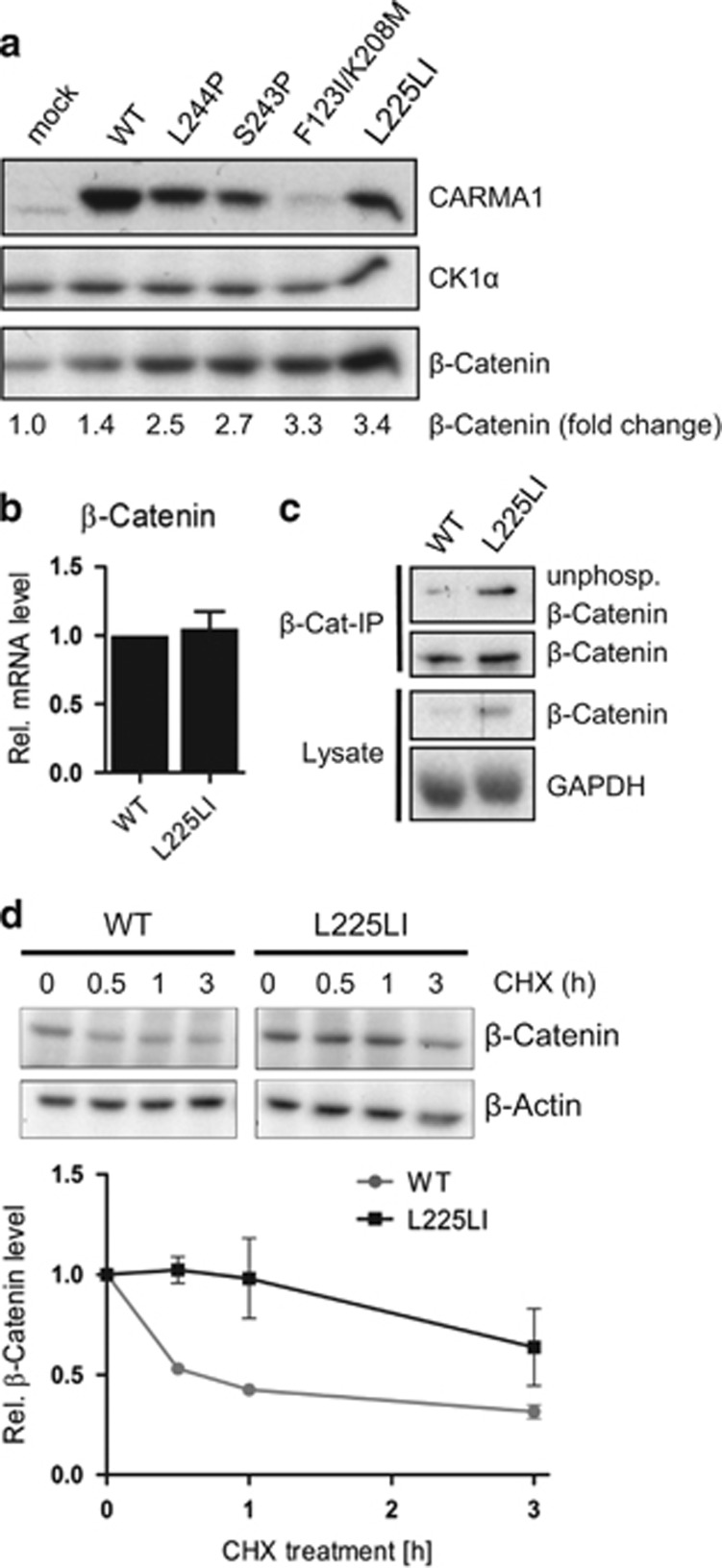
Oncogenic CARMA1 expression enhances β-catenin protein stability in BJAB cells. (**a**) β-catenin protein levels are enhanced in oncogenic CARMA1-expressing BJAB cells. Cell lysates were analyzed in western blot. Numbers indicate fold change of β-catenin normalized to CK1α in relation to mock. (**b**) β-catenin mRNA is not regulated in BJAB transduced with oncogenic CARMA1. Relative β-catenin mRNA level of WT and L225LI-transduced BJAB were determined in qRT–PCR (mean±s.e.m.; *n*=5). (**c**) Active β-catenin levels are enhanced in CARMA1 L225LI-transduced BJAB. Applied total β-catenin (βCat) amounts were adjusted after β-catenin IP and stained for unphosphorylated β-catenin in western blot. (**d**) β-catenin is stabilized in BJAB transduced with oncogenic CARMA1. CARMA1 WT and L225LI-transduced BJAB were treated with cycloheximid (CHX25 μg/ml) for indicated time points. β-catenin protein levels were assessed in western blot and normalized to β-Actin. Graph shows relative β-catenin levels normalized to β-Actin after CHX-treatment compared with untreated (0 min; *n*=2).

Unphosphorylated β-catenin was detected in CARMA1 L225LI precipitations, indicating that active CARMA1 may also exert effects on the WNT/β-catenin pathway (Figure 1f). In comparison with mock or CARMA1 WT cells, all BJAB cells expressing oncogenic CARMA1 mutants contained increased β-catenin protein amounts (Figure 2a) that were not due to enhanced mRNA expression (Figure 2b), but resulted from an accumulation of unphosphorylated β-catenin (Figure 2c). Pulse-chase experiments after cycloheximide treatment revealed stabilized β-catenin protein in the presence of CARMA1 L225LI (Figure 2d). Thus, the interaction study defined an association of oncogenic CARMA1 and β-catenin destruction complex that results in stabilization and enhanced expression of β-catenin.

### CK1α bridges the β-catenin destruction complex to CARMA1 independent of BCL10 and NF-κB activation

**Figure 3:**
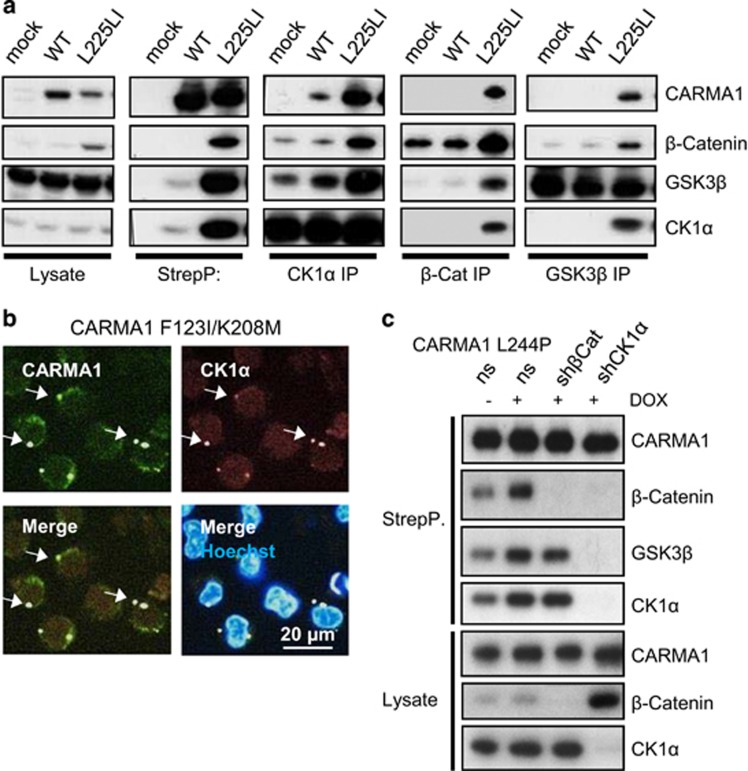
Direct interaction of CK1α to oncogenic CARMA1 results in the stabilization of β-catenin. (**a**) Reciprocal IP confirms the interaction of oncogenic CARMA1 L225LI to the β-catenin destruction complex. Endogenous IPs against CK1α, β-catenin and GSK3β in BJAB transduced with mock, CARMA1 WT or L225LI were analyzed in comparison with StrepTactin pull-down in western blot. (**b**) CK1α co-localizes with oncogenic CARMA1 F123I/K208M in the cytoplasm as shown by indirect immunofluorescence of endogenous CK1α (red, arrow) and overexpressed StrepII-CARMA1 (green, arrow). Cell nuclei were stained with Hoechst33342. CARMA1-CK1α co-aggregates are marked by arrows. (**c**) CK1α acts as a bridging factor of oncogenic CARMA1 and the β-catenin destruction complex. BJAB CARMA1 L244P cells were transduced with a DOX-inducible shRNA system containing non silencing (ns) or shRNA against β-catenin (shβCat) or CK1α (shCK1α). Following CARMA1 StrepTactin pull-down, association of the β-catenin destruction complex was monitored in western blot. Protein depletion was analyzed from cell lysates.

It has been reported previously that CK1α binds to CARMA1 and regulates the CBM complex in activated T cells and ABC DLBCL.^21^ However, the recruitment of other WNT signaling components was unexpected and we therefore verified the interaction to CARMA1 L225LI by reciprocal IP of endogenous CK1α, β-catenin and GSK3β (Figure 3a). In all instances, strong binding to CARMA1 L225LI but no or very weak association with CARMA1 WT was observed. Confocal immunofluorescence microscopy revealed that endogenous CK1α was evenly distributed in BJAB cells transduced with mock or CARMA1 WT constructs (Supplementary Figure S3a). However, CK1α co-localized strongly in CARMA1 F123I/K208M containing cytoplasmic aggregates and also with aggregates forming mutants CARMA1 L244P and S243P (Figure 3b and Supplementary Figure S3a). To investigate whether CK1α could function as the bridging factor to recruit the entire β-catenin destruction complex to CARMA1, we knocked down CK1α by a doxycyclin (DOX)-inducible lentiviral small hairpin RNA (shRNA) delivery system in BJAB cells. As expected, CK1α acts as a negative regulator of β-catenin stability and its depletion promoted strong accumulation of β-catenin in all BJAB cells (Supplementary Figure S3b). CARMA1 L225LI expression was severely diminished after CK1α knockdown, suggesting that CK1α is maintaining stability of this oncogenic mutant (Supplementary Figures S3b and c). However, expression of other CARMA1 mutants was almost unaffected by decreased CK1α levels. Indeed, binding of β-catenin and GSK3β to CARMA1 L244P was lost upon CK1α depletion, whereas the association of CK1α and GSK3β was not affected by β-catenin knockdown (Figure 3c). Thus, CK1α bridges oncogenic CARMA1 to the β-catenin destruction complex.

**Figure 4:**
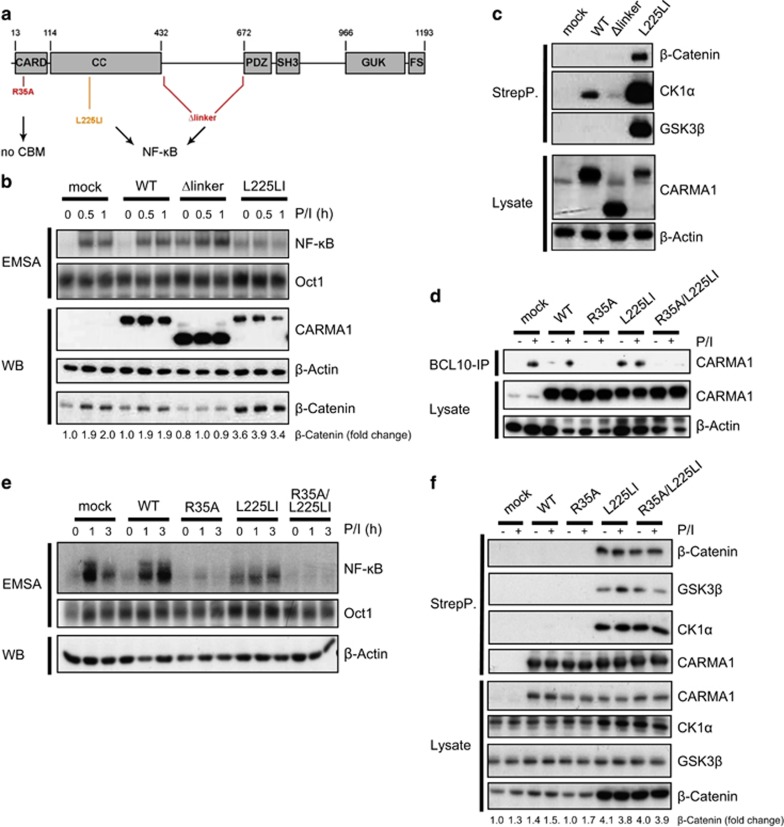
CK1α-GSK3β-β-catenin recruitment to CARMA1 is independent of BCL10 and NF-κB. (**a**) Schematic depiction of CARMA1 domains and generated mutants. CARMA1 R35A represents a BCL10-binding mutant, whereas CARMA1 Δlinker (Δ441-668), as well as GCB DLBCL-derived mutation L225LI in the CC domain activate NF-κB constitutively. (**b**) CARMA1 L225LI and Δlinker activate NF-κB constitutively. CARMA1 mutant-transduced BJAB cells were stimulated for indicated time points. NF-κB DNA binding was monitored in EMSA. Oct1 EMSA was performed for control. Numbers indicate fold change of β-catenin normalized to β-Actin in relation to mock. (**c**) CARMA1 Δlinker does not interact with the β-catenin destruction complex and does not induce β-catenin stabilization. Complex formation was monitored in western blot after StrepTactin pull-down of CARMA1 mutants in transduced BJAB cells. (**d**) CARMA1 R35A impairs inducible and constitutive BCL10 recruitment. Transduced BJAB cells were stimulated with P/I (30 min) as indicated. CARMA1 recruitment to BCL10 was analyzed in western blot after BCL10 IP. (**e**) CARMA1 R35A impairs inducible and constitutive NF-κB activation. Transduced BJAB cells were stimulated for indicated time points and NF-κB DNA binding was assessed in EMSA. Oct1 EMSA was performed for control. (**f**) Oncogenic CARMA1 associates with the β-catenin destruction complex independently of BCL10 binding. BJAB transduced with different CARMA1 constructs were stimulated with P/I (30 min) as indicated. Following CARMA1 StrepTactin pull-down, a subsequent analysis of binding partners was monitored in western blot. Numbers indicate fold change of β-catenin normalized to GSK3β in relation to mock.

To investigate more closely the role of the CBM complex and NF-κB signaling pathways downstream of oncogenic CARMA1 L225LI for β-catenin recruitment and stabilization, we examined the effects of a CARMA1 deletion mutant (CARMA1 Δlinker) that drives NF-κB signaling through the removal of the negative regulatory linker region (Figure 4a).^22^ Expression of CARMA1 Δlinker triggered NF-κB activation (Figure 4b). To address if CARMA1 Δlinker possesses oncogenic properties, we transduced the CD79B mutant ABC DLBCL cell line HBL1 that is sensitive to BTK inhibitor ibrutinib.^23^ Only oncogenic CARMA1 L225LI, but not CARMA1 Δlinker protected the ABC DLBCL cell line HBL1 from ibrutinib-induced toxicity, revealing that the Δlinker mutation can induce NF-κB, but cannot fully act like an oncogenic CARMA1 mutant (Supplementary Figures S4a–c). In line with a requirement of the CARMA1 linker region for recruitment of CK1α, the β-catenin destruction complex was not recruited to CARMA1 Δlinker (Figure 4c and Supplementary Figure S4d).^21^ Moreover, β-catenin was not stabilized revealing that strong NF-κB activation independent of CK1α binding is not promoting the effect on β-catenin (Figure 4b).

To determine whether CBM complex formation and NF-κB signaling downstream of oncogenic CARMA1 L225LI is at all required for the recruitment and stabilization of β-catenin, we generated the point mutation R35A in the CARD domain (Figure 4a).^24^ Mutation of R35A completely abolished inducible and constitutive BCL10 recruitment and NF-κB activation in the context of CARMA1 WT or CARMA1 L225LI, respectively (Figures 4d and e). However, CARMA1 double mutant R35A/L225LI was recruiting CK1α, GSK3β and β-catenin. Here β-catenin was stabilized even when binding to BCL10 was abolished (Figure 4f). To rule out that the β-catenin destruction complex is only associating with oncogenic CARMA1 upon overexpression, we performed StrepTactin pull-down in transduced HBL1 cells that express CARMA1 constructs at endogenous levels (Supplementary Figure S4d). Again, CK1α, GSK3β and β-catenin are binding to oncogenic CARMA1 L225LI in the absence or presence of BCL10 interaction (R35A/L225LI), but not to CARMA1 Δlinker. The data demonstrate that interaction of oncogenic CARMA1 to the β-catenin destruction complex does not rely on CBM complex assembly and NF-κB downstream signaling.

### High expression of β-catenin in a subset of ABC DLBCL is not required for CARMA1—NF-κB survival signaling

**Figure 5:**
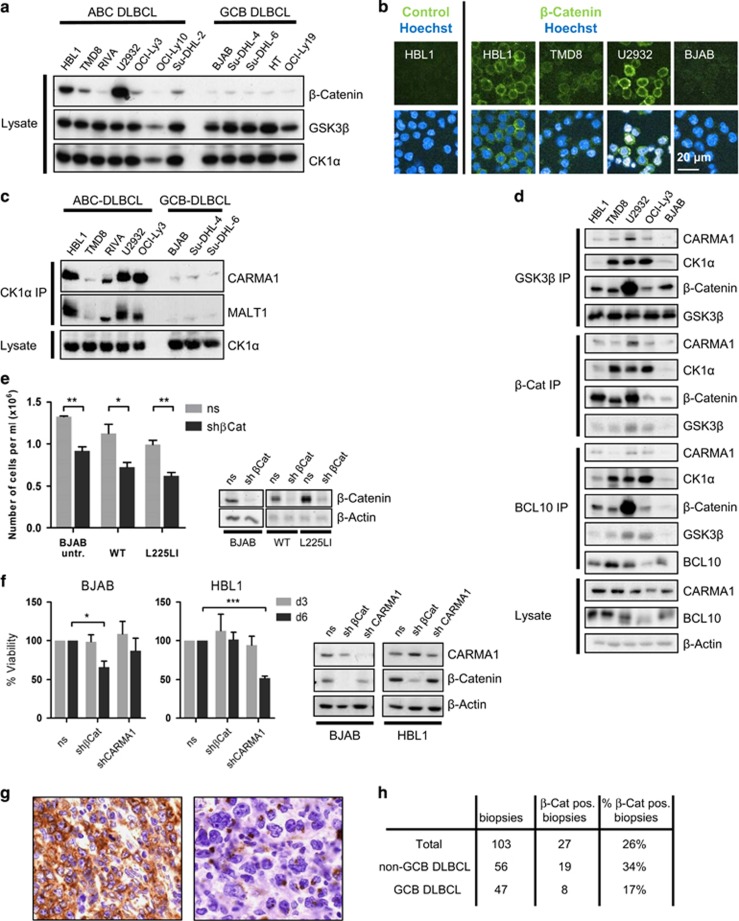
High expression of β-catenin in ABC DLBCL is not connected to tumor cell viability. (**a**, **b**) High expression of β-catenin is often found in ABC DLBCL cell lines. β-catenin protein expression was analyzed in western blot (**a**) or in indirect β-catenin immunofluorescence staining (**b**). Staining was controlled by omission of primary β-catenin antibody (left). (**c**, **d**) CARMA1 interacts with the β-catenin destruction complex in ABC DLBCL cell lines. Endogenous IPs against CK1α (**c**) or β-catenin, GSK3β and BCL10 (**d**) in DLBCL cell lines were analyzed by western blot. (**e**) β-catenin acts as a general survival factor in BJAB. BJAB cells were transduced with a DOX-inducible shRNA system containing ns or shβCat. β-catenin knockdown was verified in western blot (right). Number of viable cells by trypan blue exclusion was determined 4 days after seeding the cells at 1 × 10^5^ cells/ml in the presence of DOX (mean±s.e.m.; *n*=3). (**f**) β-catenin is not driving survival in HBL1 cells. BJAB and HBL1 were transduced with a DOX-inducible shβCat or shCARMA1. Knockdown was confirmed by western blot. Cell viability was determined in cell counts with trypan blue exclusion after 3 and 6 days of culture (mean±s.e.m.; *n*=3). (**g**) IHC of β-catenin in DLBCL as exemplified in two non-GCB DLBCL sections. (**h**) DLBCL were grouped into GCB and non-GCB subtypes using immunophenotyping according to the Hans algorithm. Positive stainings for β-catenin was over-represented in non-GCB DLBCL (one-tailed Fishers' exact test *P*=0.042).

We observed a stabilization of β-catenin upon P/I stimulation even in mock and CARMA1 WT-transduced BJAB cells, suggesting that stimulation-dependent recruitment of the β-catenin destruction complex could exert an effect equivalent to an oncogenic CARMA1 mutation (Figure 4b). Also, CK1α recruitment to CARMA1 has been demonstrated in response to antigenic stimulation in T cells and ABC DLBCL cells.^21^ We compared β-catenin expression in a panel of DLBCL cell lines to determine whether stabilization of β-catenin is a more general feature and if there may be a correlation to ABC or GCB DLBCL subtype. Whereas all GCB and two ABC (RIVA, OCI-Ly10) DLBCL cell lines showed low β-catenin expression, most ABC DLBCL cell lines showed slightly (OCI-Ly3, Su-DHL-2) to severely (TMD8, HBL1, U2932) increased β-catenin expression (Figure 5a). Confocal immunofluorescence microscopy confirmed elevated β-catenin in TMD8, HBL1 and U2932 cells and showed that β-catenin is predominately localized in the cytoplasm (Figure 5b). To address, if endogenous CARMA1 interacted with the β-catenin destruction complex in ABC DLBCL cell lines, we performed co-IPs. As observed earlier, CK1α associated with CARMA1 and MALT1 in ABC DLBCL cells (Figure 5c).^21^ Further, in all ABC DLBCL cells containing elevated β-catenin protein expression, CARMA1 was precipitated after anti-GSK3β or anti-β-catenin IP. Vice versa, BCL10 IP revealed binding of CK1α, GSK3β and β-catenin to the CBM complex in the ABC DLBCL cell lines HBL1, TMD8, U2932 and OCI-Ly3, but not in the GCB DLBCL cell line BJAB (Figure 5d). As only OCI-Ly3 cells carry a CARMA1 mutation (L244P), these data indicate that other events that are putatively upstream of the CBM complex can also trigger CARMA1-CK1α interaction and β-catenin stabilization in ABC DLBCL cells.

We next assessed whether upregulation of β-catenin could promote CARMA1-dependent viability in BJAB cells and performed knockdown of β-catenin using DOX-inducible lentiviral shRNAs. DOX treatment led to a strong reduction in β-catenin in untransduced, CARMA1 WT and CARMA1 L225LI-expressing cells (Figure 5e). β-catenin depletion resulted in impaired cell growth independent of CARMA1 expression or mutation, revealing that β-catenin promotes viability of BJAB cells (Figure 5e). To determine whether enhanced β-catenin expression is involved in CARMA1—NF-κB triggered survival signaling in ABC DLBCL cells, we knocked down β-catenin or CARMA1 in HBL1 and BJAB cells and counted the number of viable cells (Figure 5f). As expected, CARMA1 knockdown was toxic to HBL1 but not BJAB cells. β-catenin knockdown did not decrease HBL1 viability, but again affected BJAB cells to some degree. We further confirmed that β-catenin is not required for ABC DLBCL survival using a retroviral shRNA delivery system for β-catenin knockdown in HBL1, OCI-Ly3 and OCI-Ly10 cells. Transduction resulted in a robust decrease in β-catenin protein amounts in all cell lines, but cell survival was not significantly impaired (Supplementary Figures S5a and b). Also, β-catenin knockdown did not alter the sensitivity of HBL1 cells to ibrutinib-induced killing (data not shown). Thus, despite the fact that β-catenin is maintaining viability of BJAB, the data show that augmented β-catenin expression was not required for CARMA1-dependent survival signaling.

We performed immunohistochemistry on 103 primary DLBCL tumors and found β-catenin expression in 27 of 103 (Figure 5g). In most stainings, β-catenin was either diffusely expressed in the cytoplasm or recruited to subcellular cytoplasmic Golgi-like zones. Upon subclassification into GCB and non-GCB DLBCL subtypes according to the Hans classificator,^25^ 34% of non-GCB DLBCL and 17% of GCB DLBCL were positive for β-catenin, indicating that enhanced β-catenin expression is more frequent in non-GCB DLBCL (*P*=0.042, Fishers' exact test) that are over-represented in ABC DLBCL (Figure 5h). Thus, enhanced β-catenin expression is more frequently found in ABC DLBCL cell lines and non-GCB DLBCL biopsies.

### β-catenin stabilization does not suffice to induce WNT signature genes, but augments TCF/LEF activation

**Figure 6:**
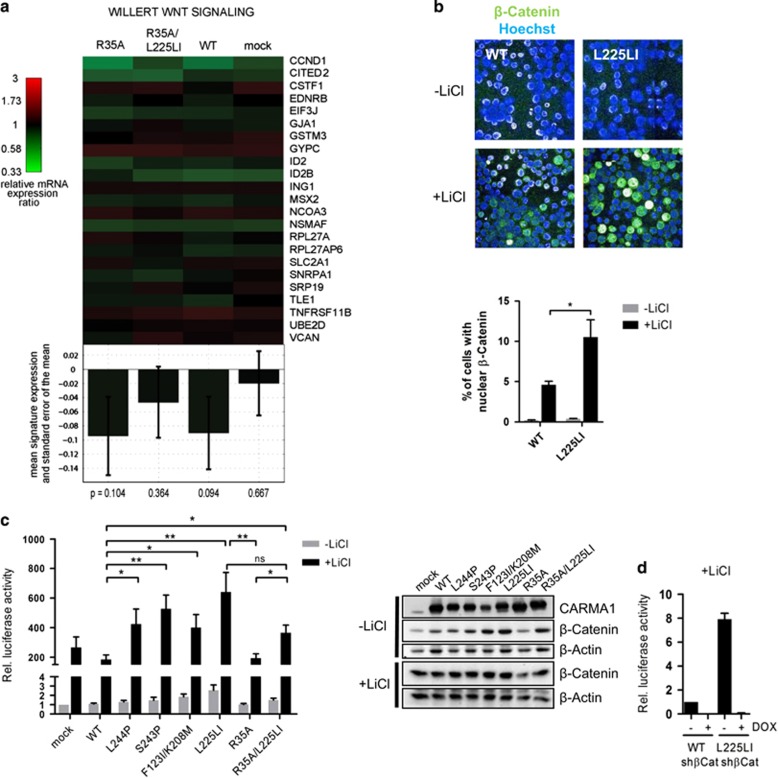
β-catenin stabilization by oncogenic CARMA1 augments TCF/LEF activation. (**a**) WNT gene signatures are not significantly regulated in transduced BJAB cells. WNT gene signatures by microarray expression profiling were compared in R35A, R35A/L225LI, WT and mock transduced BJAB cells in reference to L225LI-transduced cells. *P*-values are based on Student's *t*-tests against untransduced BJAB and error bars depict s.e.m.s. In gene set enrichment analyses, enrichment score (ES) was calculated to 0.33, *P*=0.627 via permutation test. (**b**) Induced β-catenin nuclear translocation is enhanced in BJAB CARMA1 L225LI. Transduced BJAB cells were stimulated with LiCl overnight. β-catenin localization was monitored in confocal immunofluorescence microscopy and the percentage of cells with nuclear β-catenin was quantified (mean±s.e.m.; *n*=3). (**c**) Induced TCF/LEF reporter activity is enhanced in BJAB-expressing oncogenic CARMA1. BJAB cells expressing different CARMA1 mutants were transduced with a TCF/LEF luciferase reporter. Cells were stimulated with LiCl overnight as indicated and relative TCF/LEF-regulated luciferase activity was determined (mean±s.e.m.; *n*=5). Protein levels were analyzed by western blot. (**d**) TCF/LEF reporter activity depends on β-catenin. CARMA1 WT or L225LI-expressing BJAB cells were transduced with a DOX-inducible shRNA system targeting β-catenin. Cells were treated with LiCl overnight. Relative TCF/LEF-regulated luciferase activity was determined (mean±s.e.m.; *n*=3).

We performed global gene expression profiling to elucidate if enhanced β-catenin expression by oncogenic CARMA1 could also impact on WNT and TCF/LEF signature genes. For this, we compared the gene expression of mock, CARMA1 WT, R35A, L225LI and R35A/L225LI-transduced BJAB cells. As expected expression of CARMA1 L225LI leads to a significant upregulation of the NF-κB gene signature as exemplified by an ABC DLBCL or BCR signaling-derived NF-κB signature (Supplementary Figures S6a and b).^3, 26^ NF-κB signatures were significantly lower in CARMA1 WT, R35A or L225LI/R35A-expressing cells, revealing that the recruitment of BCL10 is essential for CARMA1-triggered NF-κB activation (Supplementary Figures S6b and c). However, we did not observe significant induction or repression of other gene signatures by oncogenic CARMA1 L225LI that would be indicative of an oncogenic L225LI effect independent of NF-κB. Most importantly, we analyzed whether signatures of typical WNT-induced TCF/LEF target genes were regulated in CARMA1 L225LI and CARMA1 R35A/L225LI-expressing BJAB. We did not observe any significant alterations in the regulation of TCF/LEF gene signatures in CARMA1 L225LI or L225LI/R35A compared with mock or CARMA1 WT cells (Figure 6a and Supplementary Figure S7a).^27, 28^ Also gene set enrichment analyses confirmed significant enrichment of a representative NF-κB gene signature, but not of 12 publically available WNT gene signatures (Supplementary Figures S6d and S7b and Supplementary Table S2). Thus, β-catenin stabilization by oncogenic CARMA1 alone is apparently not sufficient to induce expression of WNT stimulated TCF/LEF target genes in BJAB cells.

As β-catenin was primarily residing in the cytoplasm of ABC DLBCL cell lines and tumor biopsies, we determined the localization of β-catenin in BJAB cells expressing CARMA1 L225LI (Figure 6b). Again, β-catenin was found predominantly in the cytoplasm. As cytoplasmic β-catenin is also involved in controlling cell adhesion by regulating the dynamic association of E-cadherin to the cytoskeleton,^29^ we monitored E-cadherin localization in immunofluorescence staining of BJAB cells transduced with CARMA1 WT or L225LI in liquid culture and upon colony formation in collagen gels (Supplementary Figures S7c and d). E-cadherin was scattered in the cytoplasm, but no differences in E-cadherin amounts or localization were observed in CARMA1 WT or L225LI-expressing cells, indicating that changes in β-catenin expression are not modulating E-cadherin-dependent processes.

As β-catenin translocates to the nucleus to act as a co-activator for the transcription factor TCF/LEF, we assessed whether elevated β-catenin levels may contribute to TCF/LEF transcriptional activation in response to WNT signaling. LiCl mimics WNT signaling and acts as a potent inducer of TCF/LEF-dependent transcription by inhibiting GSK3 activity and stabilizing β-catenin.^30^ We observed enhanced nuclear accumulation of β-catenin after LiCl treatment of CARMA1 L225LI-transduced BJAB cells when compared with CARMA1 WT cells (Figure 6b). To directly determine effects on TCF/LEF driven transcription, we transduced CARMA1-expressing BJAB cells with a TCF/LEF luciferase reporter construct (Supplementary Figure S8a).^31, 32^ Equivalent virus integration was assessed by quantitative PCR of genomic viral mCherry DNA and transduced cells were monitored based on mCherry co-expression (Supplementary Figures S8b and c). Oncogenic CARMA1 mutants, but not CARMA1 R35A/L225LI slightly enhanced mCherry expression, indicating NF-κB-dependent regulation of the SV40 promoter. In agreement with the predominant cytosolic localization, increased β-catenin expression in oncogenic CARMA1-expressing BJAB cells was hardly inducing basal transcriptional activation of TCF/LEF and only the CARMA1 L225LI mutant showed a slight increase in TCF/LEF reporter (Figure 6c). Nevertheless, TCF/LEF activity was augmented in all BJAB cells expressing oncogenic CARMA1 after LiCl treatment. These effects did not rely on NF-κB activation, because enhanced TCF/LEF transcription was not significantly impaired in the BCL10-binding mutant CARMA1 R35A/L225LI. Further, TCF/LEF reporter activity was lost upon β-catenin knockdown (Figure 6d), revealing that increased β-catenin in oncogenic CARMA1-expressing cells allows a more robust TCF/LEF transcriptional response upon activation of WNT signaling.

### Elevated β-catenin controls transcriptional activation of distinct NF-κB target genes by oncogenic CARMA1

**Figure 7:**
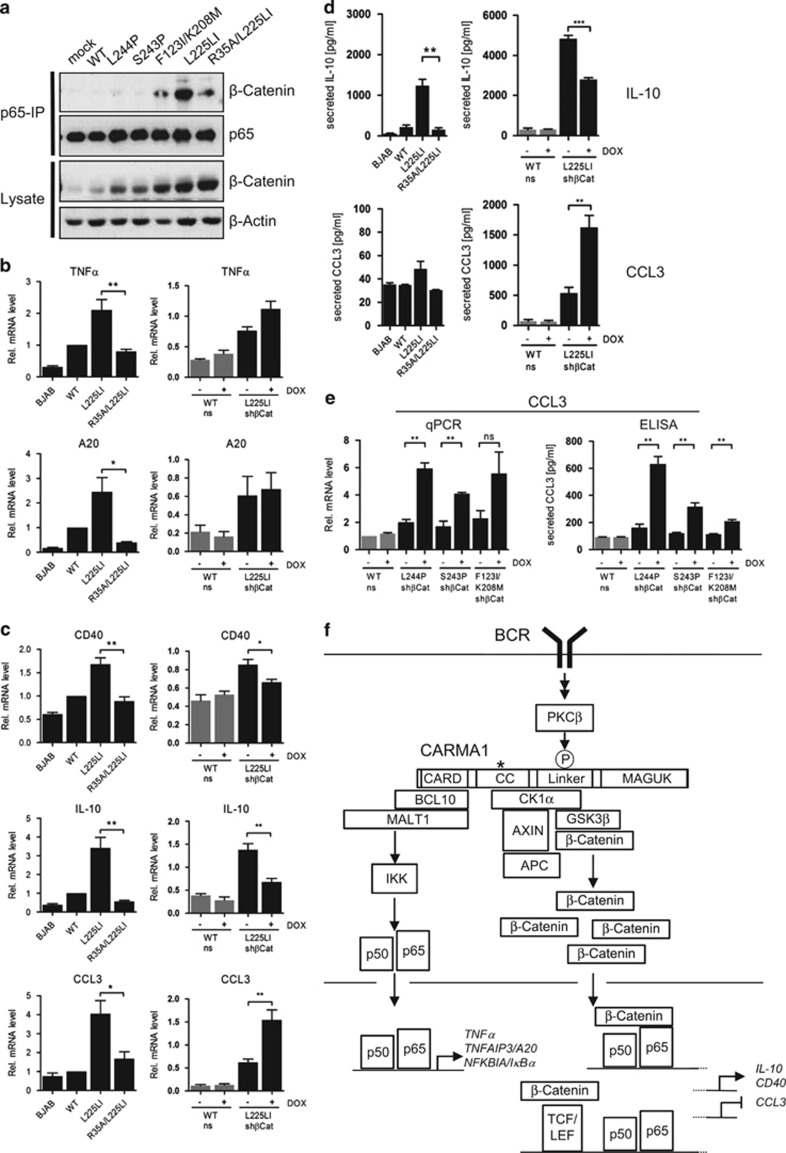
β-catenin influences the expression of distinct NF-κB target genes. (**a**) β-catenin interacts with p65 in BJAB-expressing oncogenic CARMA1. β-catenin–p65 association in transduced BJAB cells was assessed after p65 IP and western blot analyses. (**b**, **c**) Expression of distinct NF-κB target genes is regulated by β-catenin. Left: mRNA levels of NF-κB target genes in untransduced BJAB, BJAB-expressing CARMA1 WT, L225LI or R35A/L225LI were measured in qRT–PCR. Values were normalized to CARMA1 WT. Right: BJAB cells expressing CARMA1 WT or L225LI were transduced with a DOX-inducible shRNA system containing ns or shβCat. All values were related to RPII (mean±s.e.m.; *n*=4). (**d**) IL-10 and CCL3 secretion is partially influenced by β-catenin. Left: IL-10 and CCL3 secretion of untransduced BJAB, BJAB-expressing CARMA1 WT, L225LI or R35A/L225LI was determined in ELISA. Right: BJAB-expressing CARMA1 WT or L225LI were transduced with a DOX-inducible shRNA system containing ns or shβCat. Secreted IL-10 and CCL3 was measured in supernatants using ELISA (mean±s.e.m.; *n*=3). (**e**) CCL3 expression is inhibited by β-catenin in CARMA1 L244P, S243P or F123I/K208M mutant BJAB. BJAB cells were transduced with a DOX-inducible shRNA system containing ns or shβCat. Left: CCL3 mRNA expression was determined in qRT–PCR (normalized to RPII) and values were related to CARMA1 WT (mean±s.e.m.; *n*=3). Right: secreted CCL3 was measured in supernatants using ELISA (mean±s.e.m.; *n*=3). (**f**) Model for regulation of NF-κB and β-catenin cross-talk through CARMA1. Oncogenic mutations (*) or chronic BCR signaling induce parallel recruitment of BCL10 and CK1α to CARMA1. Whereas the classical CARMA1-BCL10-MALT1 (CBM) complex activates IKK/NF-κB signaling, CK1α recruits main components of the β-catenin destruction complex to CARMA1, which leads to β-catenin stabilization. Even though enhanced β-catenin expression alone is not sufficient to induce WNT signature genes, β-catenin influences expression of distinct NF-κB target genes. Whereas most genes are not influenced by β-catenin, full induction of some target genes (*CD40, IL-10*) requires β-catenin, whereas the expression of other genes is decreased by β-catenin (*CCL3*).

Previous studies have shown a physical interaction between β-catenin and NF-κB p65 in cancer cells.^33, 34^ We therefore tested if β-catenin and p65 associated in BJAB cells expressing oncogenic CARMA1 upon co-IP and found an interaction in the CARMA1 mutants F123I/K208M and L225LI-expressing BJAB cells that showed strongest β-catenin accumulation (Figure 7a). The interaction was lost in the double mutant CARMA1 R35A/L225LI, revealing that binding relies on the liberation of NF-κB/p65 from IκB proteins as well as β-catenin accumulation induced by oncogenic CARMA1.

We determined whether enhanced β-catenin amounts could affect NF-κB activation and expression of NF-κB target genes. Depletion of β-catenin by shRNA did not influence overall NF-κB DNA binding or the composition of the NF-κB complexes in CARMA1 L225LI cells (Supplementary Figure S9a). To measure effects on the expression of a panel of NF-κB target genes, we performed quantitative PCR and validated the NF-κB dependency by showing induction of multiple genes in CARMA1 L225LI but not in CARMA1 R35A/L225L-transduced cells (Figures 7b and c, Supplementary Figure S9b). Next, we tested whether the induction of these NF-κB target genes in CARMA1 L225LI cells also relied on β-catenin expression. DOX treatment triggered a severe downregulation of β-catenin mRNA expression (Supplementary Figure S9c). Expression of most genes (*TNFα*, *TNFAIP3/A20*, *ICAM1*, *NFKBIA/IκBα*, *IL-6* and *CCL4)* was not affected by the inducible β-catenin knockdown after DOX treatment (Figure 7b, Supplementary Figure S9b). However, expression of *CD40* and *IL-10* was decreased and *CCL3* was increased after β-catenin knockdown in CARMA1 L225LI cells (Figure 7c). We measured interleukin (IL)-10 and CCL3 secretion by enzyme-linked immunosorbent assay. Although high β-catenin expression was required for optimal IL-10 protein expression, downregulation of β-catenin promoted increased CCL3 secretion in CARMA1 L225LI cells (Figure 7d). To verify that β-catenin influences distinct NF-κB targets also in other CARMA1 mutants, we determined the influence of β-catenin knockdown on CCL3 and IL-10 expression in CARMA1 S243P, L244P and F123I/K208M mutant cells (Figure 7e and Supplementary Figures S9d and e). Again, high β-catenin expression was required for induction of IL-10 mRNA and protein, whereas it prevented upregulation and secretion of CCL3 in cells expressing oncogenic CARMA1 mutants. Thus, β-catenin can bind to NF-κB p65 in oncogenic CARMA1 cells and expression of β-catenin is required for the regulation of NF-κB-dependent induction of distinct genes that contribute to oncogenic properties of CARMA1.

## Discussion

Our analyses revealed a novel interaction of CARMA1 with β-catenin and its destruction complex consisting of CK1α, GSK3β, APC and AXIN that promotes β-catenin stabilization. Importantly, β-catenin binding and stabilization was independent of BCL10-MALT1 recruitment to CARMA1 and downstream NF-κB activation. Thus, oncogenic CARMA1 integrates two distinct signaling complexes, namely BCL10-MALT1 (the ‘classical' CBM) and the β-catenin destruction complex, which induces in parallel constitutive NF-κB activation and stabilization of β-catenin (Figure 7f). Even though the increase in β-catenin was not sufficient to induce WNT signature genes, WNT signaling was augmented and distinct NF-κB target genes were co-activated or repressed by β-catenin.

We defined CK1α as the bridging factor that connects the β-catenin destruction complex to CARMA1. Interaction of CK1α and CARMA1 has already been shown to contribute to NF-κB activation in T cells and ABC DLBCL.^21^ Our results demonstrate that the function of CARMA1-CK1α binding is not mono-directional and only feeding into the NF-κB pathway, but also recruits and controls β-catenin in cells carrying oncogenic CARMA1 mutations. In fact, CK1α binding and β-catenin stabilization was also detected for CARMA1 WT after P/I stimulation, which is consistent with previous reports showing β-catenin stabilization upon T-cell receptor and BCR engagement in a PKCθ- and PKCβ-dependent manner, respectively.^35, 36^ In line with BCR-dependent β-catenin stabilization, we found recruitment of the β-catenin destruction complex and elevated β-catenin expression in ABC DLBCL cell lines that display chronic BCR signaling as a result of upstream mutations, for example, in CD79B. At present, it remains unclear how β-catenin is stabilized by CARMA1, but it seems reasonable that CARMA1 recruitment triggers inactivation of the β-catenin destruction complex in a manner that is similar to Frizzled receptor association after WNT stimulation.^16, 37^ Upon WNT stimulation, the entire destruction complex including β-catenin is recruited to and inactivated at the Frizzled receptor at the membrane to prevent ubiquitin-dependent β-catenin degradation.^16, 37^ Thus, CARMA1 activation through BCR signaling or oncogenic mutations seems to stabilize β-catenin by a mechanism that is analogous to the WNT-Frizzled pathway.

As β-catenin was predominantly found in the cytosol, increased β-catenin in oncogenic CARMA1 cells was apparently not sufficient to drive strong TCF/LEF transcriptional activation or induce a WNT target gene signature. Nevertheless, we suppose that a small fraction of stabilized β-catenin is localizing in the nucleus, as we detected slightly augmented TCF/LEF reporter activation in CARMA1 L225LI cells. Importantly, elevation of β-catenin by all oncogenic CARMA1 mutants promoted TCF/LEF reporter activity after WNT pathway activation. In line with a previous report, we could detect enhanced β-catenin protein expression in a subset of DLBCL biopsies.^38^ However, Ge *et al.* found β-catenin largely in the nucleus of DLBCL cells and we detected predominantly cytosolic staining in the ABC DLBCL cell lines and DLBCL biopsies. The positive β-catenin staining in DLBCL resembled the perinuclear or diffuse localization observed in lymphocytes or chronic lymphocytic leukemia cells.^39^ We observed a significant over-representation of β-catenin positivity in non-GCB (34%) versus GCB (17%) DLBCL samples. Even though the status of CARMA1 and other oncogenes was not analyzed in the respective patient samples, high β-catenin amounts are more frequent than CARMA1 mutations and may be in the range of patients that carry either CARMA1 or CD79B mutations (~30% of all ABC DLBCL).^3, 7, 9^ Clearly, further analyses will be required to correlate the mutation status and β-catenin levels in DLBCL subtypes. The observation that a considerable number of GCB and not all non-GCB/ABC DLBCL cells were β-catenin positive indicates that alternative processes besides chronic BCR signaling may contribute to β-catenin stabilization. Indeed, WNT3a secretion by a subpopulation of cells in a DLBCL population was shown to be able to induce WNT signaling, indicating that especially in primary samples the tumor microenvironment may contribute to increasing β-catenin amounts.^40^ Thus, the increased β-catenin levels could be at least partially a result of autocrine or paracrine WNT signals within the tumor microenvironment.

Stabilized β-catenin in oncogenic CARMA1-expressing BJAB cells is interacting with NF-κB p65 and β-catenin regulates the expression of distinct NF-κB target genes. An NF-κB—β-catenin cross-talk that modulates gene expression through physical interaction has already been observed in colon and breast cancer cells.^33^ Binding of β-catenin and p65 were shown to enhance tumorigenesis also in intestinal epithelial cells.^34^ Co-regulation of target genes may either rely on the direct binding of β-catenin and p65, but it can also function more indirectly at the level of transcriptional activation. β-catenin-TCF/LEF complexes were shown to induce IL-10 transcription through binding sites in the promoter.^41^

Functionally, β-catenin knockdown did not affect CARMA1-triggered survival of different ABC DLBCL cell lines and the decreased viability upon β-catenin knockdown in BJAB cells was also not connected to oncogenic CARMA1 or the NF-κB activation status. In line with this we did not observe β-catenin co-regulation of critical antiapoptotic NF-κB target genes. Thus, β-catenin is not involved in controlling cell-intrinsic survival of DLBCL cell lines, but this may change in a setting when WNT signaling is active, for example, in a tumor microenvironment. Interestingly, NF-κB target genes that are also regulated by β-catenin are the cytokine IL-10 and the chemokine CCL3 that both have been associated with regulating cell communication and antitumor immunity. NF-κB-dependent IL-10 and IL-6 secretion can activate STAT3 signaling in an autocrine manner.^26^ As IL-6 expression was not affected by β-catenin knockdown, this interplay is most likely not severely affected. However, tumor-induced β-catenin activation and IL-10 secretion in dendritic cells can suppress CD8 T-cell-dependent antitumor immunity.^42^ Also, β-catenin-driven IL-10 production in melanoma cells impaired dendritic cell maturation and inhibited an antitumor immune response.^41^ Further, high expression of CCL3 (MIP-1α) and other chemokines in melanomas was associated with improved recruitment of CD8 effector T cells.^43^ CCL3 is a candidate for gene therapy, because its heterologous expression can enhance antitumor immune responses.^44, 45^ Recently, melanoma WNT/β-catenin signaling was shown to prevent antitumor immunity and CCL3 and CCL4 expression were repressed by high β-catenin expression.^46^ Even though we did not see effects on CCL4 production by oncogenic CARMA1 in the absence of β-catenin, these data point out that β-catenin is able to repress the expression of chemokines that promote T-cell infiltration. Thus, increased IL-10 and decreased CCL3 production in ABC DLBCL that display augmented β-catenin activation may add to an immunosuppressive tumor microenvironment. Of note, mature B-cells switch upon expression of oncogenic CARMA1 from self-antigen-induced cell death to proliferation and IκB kinase β/NF-κB signaling is not sufficient to induce this shift.^47^ It will be interesting in how far augmented β-catenin may be required in this process.

Our data using oncogenic CARMA1 mutants unravel a novel cross-talk between the tumorigenic NF-κB and β-catenin pathways. Functionally, we demonstrated that β-catenin activates or suppresses certain NF-κB-dependent cytokines and chemokines that control the tumor microenvironment and especially have been linked with preventing antitumor immunity in cancers. It will be interesting to evaluate the effects of β-catenin for tumor immunity in DLBCL. Our data suggest that the critical cross-talk of NF-κB and β-catenin could be promising for future therapeutic approaches.

## Materials and methods

### Antibodies, plasmids, shRNA

Following antibodies were used: β-catenin (clone 14), e-Cadherin (610405) (all BD); AXIN1 (S20), BCL10 (331.3, C17), β-Actin (I-19), p50 (NLS), p65 (C-20), cRel (N), CK1α (C-19) (all Santa Cruz Biotechnology, Heidelberg, Germany); CARMA1 (1D12), GSK3β (27C10), GAPDH-HRP (14C10) (all Cell Signaling, Frankfurt am Main, Germany); unphosphorylated β-catenin (05-665, Millipore, Darmstadt, Germany); StrepTagII (IBA, Göttingen, Germany), hCD2-APC (eBioscience, Frankfurt am Main, Germany), Tubulin (DM1A, Sigma, Taufkirchen, Germany); horseradish peroxidase-conjugated secondary antibodies (Jackson/Dianova, Hamburg, Germany). Following lentiviral constructs were used: pHAGE-PGK-hCD2-T2A backbone^48^ contained Flag-Strep-Strep-tagged^49^ CARMA1; Lentiviral infection, shRNA expression and TCF/LEF luciferase reporter constructs were described before.^31, 32^ shRNA sequences: CARMA1 5′-GGGAGAATGTGGAGTGTAA-3′, β-catenin (#1: 5′-AGGTGCTATCTGTCTGCTCTA-3′, #2: 5′-CGCATGGAAGAAATAGTTGAA-3′, #3: 5′-GCTTGGAATGAGACTGCTGAT-3′), CK1α 5′-GAGCAAGCTCTATAAGATTCT-3′, MSMO1 5′-CTCTCAACCCTTTAAATCTGA-3′, Myc 5′-CCTATGAACTTGTTTCAAATG-3′.

### Cell culture, cell lines, viral transduction, viability and TCF/LEF reporter assays

Unless otherwise stated, DLBCL lines were cultured in 15% fetal bovine serum and 100 U/ml penicillin/streptomycin containing Roswell Park Memorial Institute medium at 37 °C and 5% CO_2_, except OCI-Ly19 that were maintained in IMDM (Iscove's Modified Dulbecco's Medium) and OCI-Ly10 in IMDM containing 20% human serum instead. DLBCL cell lines have been obtained by DSMZ (Braunschweig, Germany) or cooperation partners. The cells are authenticated by sequencing known mutations and are checked for mycoplasms on a regular basis. For stimulations 200 ng/ml PMA and 300 ng/ml Ionomycin (Calbiochem, Darmstadt, Germany) were applied. Lentivirus and retrovirus production and transductions were described previously.^4, 50, 51^ Cells were treated with DOX (0.02 μg/ml) for 3 days to induce shRNA expression. Flow cytometry analysis was conducted using Attune Acoustic Focusing Cytometer system (Thermo Scientific, Waltham, MA, USA) and FlowJo (Treestar, Ashland, OR, USA) software. Cell viability was determined by counting cell numbers after trypan blue exclusion. TCF/LEF luciferase reporter activity was measured in a luminometer by adding Luciferase Assay ReagentII (Promega, Madison, WI, USA) to cell lysates that were adjusted using Bradford method.

### Co-immunoprecipitation, mass spectrometry and electrophoretic mobility shift assay

For immunoprecipitation studies cells were lysed in co-IP buffer (25 mM 4-(2-hydroxyethyl)-1-piperazineethanesulfonic acid pH 7.5, 150 mM NaCl, 0.2% NP-40, 10% glycerol, phosphatase inhibitors 1 mM dithiothreitol, 10 mM sodium fluoride, 8 mM β-glycerophosphate, 300 μM sodium vanadate and protease inhibitors (Roche, Grenzach, Germany)) followed by antibody incubation as described previously.^52^ For disruption of nuclei, cells were additionally passed through a 26-gauge needle. StrepTactin-PD were conducted in co-IP buffer using Strep Tactin-Sepharose (IBA). Harsh cell lysis was conducted in radioimmunoprecipitation assay buffer (0.1% sodium dodecyl sulfate, 250 mM NaCl, 10 mM Tris pH 7.4, 0.5% sodiumdeoxycholate, 1% TritonX) supplied with phosphatase and protease inhibitors. Mass spectrometry was carried out as described in the supplement. High-salt buffer lysates were analyzed in electrophoretic mobility shift assay as previously described.^53^

### Gene expression profiling by microarray

RNA was extracted using RNeasy Mini Kit (QIAGEN, Hilden, Germany) and duplicates of each sample were analyzed in genome wide expression analyses on an Illumina HT12 v4 1-color chip (BIO.LOGIS, Frankfur am Main, Germany) according to the manufacture protocol. Details are given in the supplement. The gene expression data have been deposited in the Gene Expression Omnibus database^54^ of the National Center for Biotechnology Information (www.ncbi.nlm.nih.gov/geo; accession number GSE70025).

### Quantitative real-time-PCR and enzyme-linked immunosorbent assay

Quantitative real-time-PCR was conducted as previously described.^50^ mRNA was normalized by RNA polymerase II mRNA. Primer sequences are available in Supplement. IL-10 and CCL3 secretion was measured 20 h after accumulation using human ready-set-go ELISA kit (eBioscience).

### Immunohistochemistry

Tumors from 103 untreated DLBCL patients were immunostained for CD10, BCL6 and IRF4/MUM1 on a tissue microarray format to establish GCB and non-GCB types according to the Hans classificator.^25^ β-catenin staining was done on a semi-automated LabVision tissue stainer using the monoclonal antibody clone 14 (CELL MARQUE, Rocklin, CA, USA) at a dilution of 1:25. Cases showing unequivocal staining in >30% of cells were regarded as positive. Use of archival formalin-fixed DLBCL samples was approved by the ethics committee of the University of Würzburg.

### Statistical analyses

Mass spectrometry was analyzed using analysis of variance test and microarrays were analyzed using Student's *t*-test as further specified in the supplement. Sample size is mentioned for each experiment and data are expressed as mean±s.e.m. Unless specified otherwise statistical significance was determined using unpaired Student's *t*-test with *P⩽0.05, **P⩽0.01 and ***P⩽0.001.
