# Supplementary material for: Oncogenic CARMA1 couples NF-κB and β-catenin signaling in diffuse large B-cell lymphomas
Source: Oncogene. 2016 Jan 18;35(32):4269–81. doi: 10.1038/onc.2015.493 (PMC4981874; doi:10.1038/onc.2015.493)
Supplement: Supplementary Information [file onc2015493x1.docx]

**Bognar et al.**

**SUPPLEMENTARY INFORMATION**

**SUPPLEMENTARY MATERIAL AND METHODS**

*Mass spectrometry approach*

2x 10^8^ transduced BJAB cells were lysed in co-IP buffer, 2x freeze-thaw cycles and 3 strokes in a dounce homogenizer. Following Strep-precipitation, beads were resuspended in 50 mM ammoniumbicarbonate and associated proteins were digested with 2.5 µg trypsin (Sigma) overnight at 37 °C. Peptides were measured in label-free LC-MS/MS, using an LTQ Orbitrap XL (Thermo scientific). Three independent precipitations were prepared for proteomics and significance was determined using ANOVA method as described in detail before.^1^ Briefly, relative quantification was performed based on cumulated peptide abundances of all unique peptides per protein. After normalization, statistical analysis was performed using transformed normalized abundances for one-way analysis of variance (ANOVA) calculations of all detected features and protein abundances were compared to protein abundances in precipitation control. Identified proteins were considered as specific interaction partners, if (i) the ratio of cumulated protein abundances in the sample versus mock control was above 2, (ii) the calculated ANOVA p-value was below 0.05 and (iii) the candidate was detected in at least in one sample in all three experiments. The relative mean enrichments were depicted with + = 2-10, ++ = 10-50 and +++ ≥50.

*Gene expression profiling and gene set enrichment analysis*

RNA was extracted using RNeasy Mini Kit (QIAGEN). Duplicates of each sample were analyzed in genome wide expression analyses on an Illumina HT12 v4 1-color chip (BIO.LOGICS) in reference to untransduced BJAB samples. Measured gene expressions were imported on raw bead level. All 10 microarrays were preprocessed and normalized together. Their intensity distributions for all measured beads were equalized by quantile normalization. The background mode was fitted and removed and a spot filter was applied to exclude too dim beads (< mean background intensity). Intensities were log2-transformed and aggregated by measured sequence to form beadsets. Beadsets with more than 50% of their beads excluded by the spot filter were also excluded. Further analysis was performed on gene level using median aggregation and manufacturer’s annotations. For genes having multiple beadsets, the one with the highest expression (in average over all arrays) was selected. Moreover, signals from mock, CARMA1 WT, R35A or R35A/L225LI transduced BJAB cells were compared to BJAB CARMA1 L225LI as reference. To analyze the association to NF-κB and WNT, gene set enrichment analyses for corresponding signatures from the Molecular Signature Database v3.1,^2^ the GeneSigDB v4,^3^ and from the Staudt lab library^4^ were performed as previously described.^5^ For visualization, heatmaps depict gene expressions according to the color scale shown. To assess the significance of differential expressions, signature averages were compared to zero difference via t-tests; error bars depict SEMs. The gene expression data has been deposited in the Gene Expression Omnibus (GEO) database^6^ of the National Center for Biotechnology Information (www.ncbi.nlm.nih.gov/geo; accession number GSE70025).

*Quantitative RT-PCR primer*

Following primer sequences were used in qRT-PCR: CCL3 (MIP-1α) for: 5´- GGCTCTCTGCAACCAGTTCT -3´ rev: 5´- TGAAATTCTGTGGAATCTGCC -3´; CCL4 for: 5´- CAGCGCTCTCAGCACCAA -3´ rev: 5´- AGCTTCCTCGCAGTGTAAGAAAA -3; CTNNB1 (β-Catenin) for: 5´-GCAGAACTTGCCACACG-3´ rev: 5´-GCGTGTCTGGAAGCTTCC-3´; ICAM1 for: 5´-GGCTGGAGCTGTTTGAGAAC-3´ rev: 5´-ACTGTGGGGTTCAACCTCTG-3´; IL-10 for: 5´-CTGGAGGAGGTGATGCCCCAA-3´ rev: 5`-ACCTGCTCGACGGCCTTGCT-3´ ; IL-6 for: 5´-GGTACATCCTCGACGGCATCT-3´ rev: 5´-GTGCCTCTTTGCTGCTTTCAC-3´; NFKBIA (IκBα) for: 5´-CCGCACCTCCACTCCATCC-3´ rev: 5´-ACATCAGCACCCAAGGACACC-3´; RPII for: 5´-GCACCACGTCCAATGACAT-3´ rev: 5´-GTGCGGCTGCTTCCATAA-3´; TNFAIP3 (A20) for: 5´-TTTTGTACCCTTGGTGACCCTG-3´ rev: 5´-TTAGCTTCATCCAACTTTGCGG-3´; TNFRSF5 (CD40) for: 5´-GCAGTGGGTGGTTCTGGAT-3´ rev: 5´-CTGGTCTCACCTCGCTATGG-3´; TNFα for: 5´-CCCAGGGACCTCTCTCTAATCA-3´ rev: 5´-GCTACAGGCTTGTCACTCGG-3´; mCherry for: 5´-GCTTCAAGTGGGAGCGCG-3´ rev: 5´-GGAAGTTGGTGCCGCGCAG-3´.

*Indirect immunofluorescence staining and analyses*

Cells were fixed and permeabilized on PolyD lysine (Sigma) coated glass bottom ViewPlate-96F (Perkin Elmer). Primary antibody staining was followed by secondary antibody staining with Alexa Fluor488 or Alexa Fluor633-conjugated secondary antibody (Lifetechnologies) and counterstained with Hoechst33342. 60x images were taken with an Operetta system (Perkin Elmer) and analyzed with Harmony 3.5 software. For staining of cell colonies, 1.5x 10^4^ cells/ml were seeded in collagen gels using 3D Collagen Cell culture system (Millipore). After 7 days, gels were fixed with 4% PFA and quenched with 0.15 M glycine. Immunostainings were done according to standard protocols. Gels were mounted with Aqua-Poly/Mount Coverslipping Medium (Polyscience). Samples were imaged on an inverted confocal laser scanning microscope (Olympus) with 60x magnification.

**SUPPLEMENTARY REFERENCES**

1 Hauck SM, Dietter J, Kramer RL, Hofmaier F, Zipplies JK, Amann B *et al*. Deciphering membrane-associated molecular processes in target tissue of autoimmune uveitis by label-free quantitative mass spectrometry. *Molecular & cellular proteomics : MCP* 2010; **9:** 2292-2305.

2 Liberzon A, Subramanian A, Pinchback R, Thorvaldsdottir H, Tamayo P, Mesirov JP. Molecular signatures database (MSigDB) 3.0. *Bioinformatics (Oxford, England)* 2011; **27:** 1739-1740.

3 Culhane AC, Schroder MS, Sultana R, Picard SC, Martinelli EN, Kelly C *et al*. GeneSigDB: a manually curated database and resource for analysis of gene expression signatures. *Nucleic acids research* 2012; **40:** D1060-1066.

4 Shaffer AL, Wright G, Yang L, Powell J, Ngo V, Lamy L *et al*. A library of gene expression signatures to illuminate normal and pathological lymphoid biology. *Immunological reviews* 2006; **210:** 67-85.

5 Subramanian A, Tamayo P, Mootha VK, Mukherjee S, Ebert BL, Gillette MA *et al*. Gene set enrichment analysis: a knowledge-based approach for interpreting genome-wide expression profiles. *Proceedings of the National Academy of Sciences of the United States of America* 2005; **102:** 15545-15550.

6 Barrett T, Wilhite SE, Ledoux P, Evangelista C, Kim IF, Tomashevsky M *et al*. NCBI GEO: archive for functional genomics data sets--update. *Nucleic acids research* 2013; **41:** D991-995.

**SUPPLEMENTARY FIGURE LEGENDS**

**Supp. Table 1: Mass spectrometry approach identifies a list of putative CARMA1 interaction partners.** LC-MS/MS identifies a total of 33 proteins in association to CARMA1. Three independent ST-PD experiments were conducted in CARMA1 WT, WT stimulated (30 min P/I) and four different oncogenic CARMA1 mutant transduced BJAB. Solely significant protein interaction partners are listed with the premise that they passed ANOVA test (p-value< 0.05) and were identified in all three precipitation experiments of one sample at least. The ratio of mean peptide abundances of each sample related to the mock precipitation control was calculated and depicted with + = 2-10, ++ = 10-50 and +++ = >50. Infinite stands for incalculable values, because no peptides were detected in mock control. ANOVA p-values are given for each identified protein calculated in relation to mock control. † marks proteins that were identified by one single peptide.

**Supp. Table 2: WNT gene signatures are not significantly changed in BJAB cells expressing CARMA1 mutants.** Gene set enrichment analyses of 12 publically available WNT signatures were based on the comparison of BJAB transduced with CARMA1 R35A, R35A/L225LI, WT, mock versus BJAB CARMA1 L225LI. p-values were calculated in permutation test.

**Supp. Figure 1: Transduction of CARMA1 in BJAB cells generates a polyclonal cell pool.** Schematic depiction of CARMA1 domain structure and mutants. CARMA1 proteins were co-expressed with the cellular surface marker hΔCD2 (human∆CD2), which was separated from CARMA1 protein via a ribosomal skip mechanism induced by a T2A sequence. h∆CD2 expression was analyzed by flow cytometry after staining with an APC-coupled antibody.

**Supp. Figure 2: CARMA1 mutants are recruited into insoluble aggregates.** (a) CARMA1 constructs are expressed equally in transduced BJAB cells. Transduced BJAB cells were lysed with RIPA or co-IP buffer and protein levels were analyzed in Western blot. CARMA1 levels were quantified using β-Actin as loading control and related to mock transduced BJAB cells. (b and c) CARMA1 mutants are recruited into subcellular aggregates. (b) Representative immunofluorescence images of StrepII-tag stained CARMA1 in transduced BJAB cells. (c) Aggregates were quantified and related to BJAB CARMA1 WT (mean ± SEM; n=3).

**Supp. Figure 3: CK1α acts as bridging factor for the association of the β-Catenin destruction complex to oncogenic CARMA1.** (a) CK1α co-localizes with oncogenic CARMA1 in the cytoplasm of transduced BJAB cells as shown in parallel immunofluorescence staining of endogenous CK1α and overexpressed StrepII-CARMA1. Cell nuclei were counterstained with Hoechst33342. (b and c) CK1α depletion influences CARMA1 L225LI stability. BJAB cells expressing CARMA1 WT or CARMA1 mutants were transduced with a DOX inducible shRNA system containing non-silencing (ns) or shRNA against β-Catenin (shβCat) or CK1α. (b) Cell lysates were analyzed in Western blot. (c) ST-PD of CARMA1 L225LI was assessed in Western blot after β-Catenin or CK1α depletion.

**Supp. Figure 4: Stable expression of oncogenic CARMA1 to endogenous levels induces association of the β-Catenin destruction complex in HBL1 cells.** (a) Transduction of CARMA1 in HBL1 cells results in a polyclonal cell pool. h∆CD2 co-expression was determined in flow cytometry after staining with an APC-coupled antibody. (b) CARMA1 expression in transduced HBL1 cells was analyzed by Western blot. (c) CARMA1 L225LI but not CARMA1 ∆linker expression protects HBL1 cells from toxicity induced by BTK inhibition. HBL1 cells were transduced with CARMA1 constructs and treated with Ibrutinib (2nM) for 4 days. Detection of viable HBL1 cells are given in relation to DMSO treated control (mean ± SEM; n=3). (d) Oncogenic CARMA1 associates with the β-Catenin destruction complex in transduced HBL1 cells. Complex formation was monitored in Western blot after ST-PD of CARMA1 mutants in transduced BJAB cells.

**Supp. Figure 5: High expression of β-Catenin in ABC DLBCL is not connected to tumor cell viability.** (a and b) HBL1, OCI-Ly3 and OCI-Ly10 ABC DLBCL cells were transduced with a retroviral shRNA delivery system containing three independent shRNAs directed against βCat. (a) β-Catenin knock-down was verified in Western blot. shRNA directed against MSMO1 served as a control. (b) Cell viability was determined in cell counts with trypan blue exclusion (mean ± SEM; n=3). Positive control shRNA directed against MYC was toxic to all DLBCLs.

**Supp. Figure 6: Stable expression of oncogenic CARMA1 in BJAB cells induces constitutive NF-κB activation.** (a) NF-κB target gene expression is significantly enhanced in BJAB expressing CARMA1 L225LI. A representative NF-κB signature is shown for two independent RNA replicates of BJAB CARMA1 L225LI in reference to untransduced BJAB cells. Enrichment score (ES) -0.71, p<=0.001 via permutation test. (b-d) Upregulation of NF-κB signatures depends on active NF-κB signaling. Gene signatures of CARMA1 R35A, R35A/L225LI, WT or mock transduced BJAB sample RNA were compared to CARMA1 L225LI. p-values are based on Student t-tests against untransduced BJAB and error bars depict SEMs. ES 0.56, p<=0.001 (b) and ES 0.75, p<=0.001 (c). (d) Gene set enrichment analyses of NF-κB gene signature shown in (c).

**Supp. Figure 7: β-Catenin stabilization does not alter β-Catenin dependent processes in BJAB expressing oncogenic CARMA1.** (a and b) WNT gene signatures are not significantly regulated in transduced BJAB cells. (a) WNT gene signatures profiles were compared in R35A, R35A/L225LI, WT and mock transduced BJAB cells in reference to L225LI transduced cells. Left: ES 0.26, p=0.613, Right: ES -0.202, p=0.975. (b) Gene set enrichment analyses of WILLERT WNT SIGNALING gene signature based on comparison of BJAB transduced with CARMA1 R35A, R35A/L225LI, WT, mock versus BJAB CARMA1 L225LI. (c and d) β-Catenin stabilization is not affiliated with altered E-Cadherin localization or expression. Representative pictures of indirect immunofluorescence staining of endogenous E-Cadherin in CARMA1 WT or L225LI transduced BJAB grown in liquid culture (c) or in collagen gel (d). Nuclei were counterstained with Hoechst33342.

**Supp. Figure 8: TCF/LEF reporter is equally transduced into BJAB cells.** (a) Schematic depiction of TCF/LEF reporter construct. 7xTcf-Luciferase reporter cassette is followed by an SV40-mCherry cassette. (b) Lentiviral TCF/LEF reporter transduction results in equal integration into BJAB cells expressing CARMA1. Integration levels of TCF/LEF reporter were determined using genomic DNA extractions of transduced BJAB cells. qRT-PCR were performed using primers directed against mCherry and values were related to RPII (mean ± SEM; n=3). (c) Transduction of TCF/LEF reporter in BJAB cells results in a polyclonal cell pool. mCherry expression was determined in flow cytometry.

**Supp. Figure 9: β-Catenin influences the expression of distinct NF-κB target genes.** (a) β-Catenin does not influence NF-κB DNA binding. BJAB expressing CARMA1 L225LI were transduced with a DOX inducible shRNA system containing shRNA against shβCat and analyzed by EMSA after DOX treatment. Supershift EMSA was performed using antibodies specific for p50, p65 and cRel and shifted complexes are marked with an asterisk (*). Positions of p50/p50, p50/cRel and p50/p65 NF-κB complexes are indicated by arrows. Oct1 EMSA was performed for control. Protein expression was assessed in Western blot analyses. (b) Expression of distinct NF-κB target genes is partially influenced by β-Catenin. Left: mRNA levels of NF-κB target genes in untransduced BJAB or BJAB expressing CARMA1 WT, L225LI or R35A/L225LI were measured in qRT-PCR. Values were normalized to CARMA1 WT (mean ± SEM; n=4). Right: BJAB cells expressing CARMA1 WT or L225LI were transduced with a DOX inducible shRNA system containing ns or shβCat (mean ± SEM; n=3). qRT-PCR was normalized to RPII. (c) β-Catenin depletion was verified in RT-qPCR (mean ± SEM; n=3). (d) BJAB cells expressing CARMA1 were transduced with a DOX inducible shRNA system containing ns or shβCat. β-Catenin knock down was assessed in Western blot. (e) IL10 expression is enhanced by β-Catenin in CARMA1 L244P, S243P or F123I/K208M mutant BJAB. BJAB cells were transduced with a DOX inducible shRNA system containing ns or shβCat. Left: IL10 mRNA expression was determined in qRT-PCR (normalized to RPII) and values were related to CARMA1 WT (mean ± SEM; n=3). Right: Secreted IL10 was measured in supernatants using ELISA (mean ± SEM; n=3).
