# Supplementary figures and images for: Oncogenic CARMA1 couples NF-κB and β-catenin signaling in diffuse large B-cell lymphomas
Source: Oncogene. 2016 Jan 18;35(32):4269–81. doi: 10.1038/onc.2015.493 (PMC4981874; doi:10.1038/onc.2015.493)

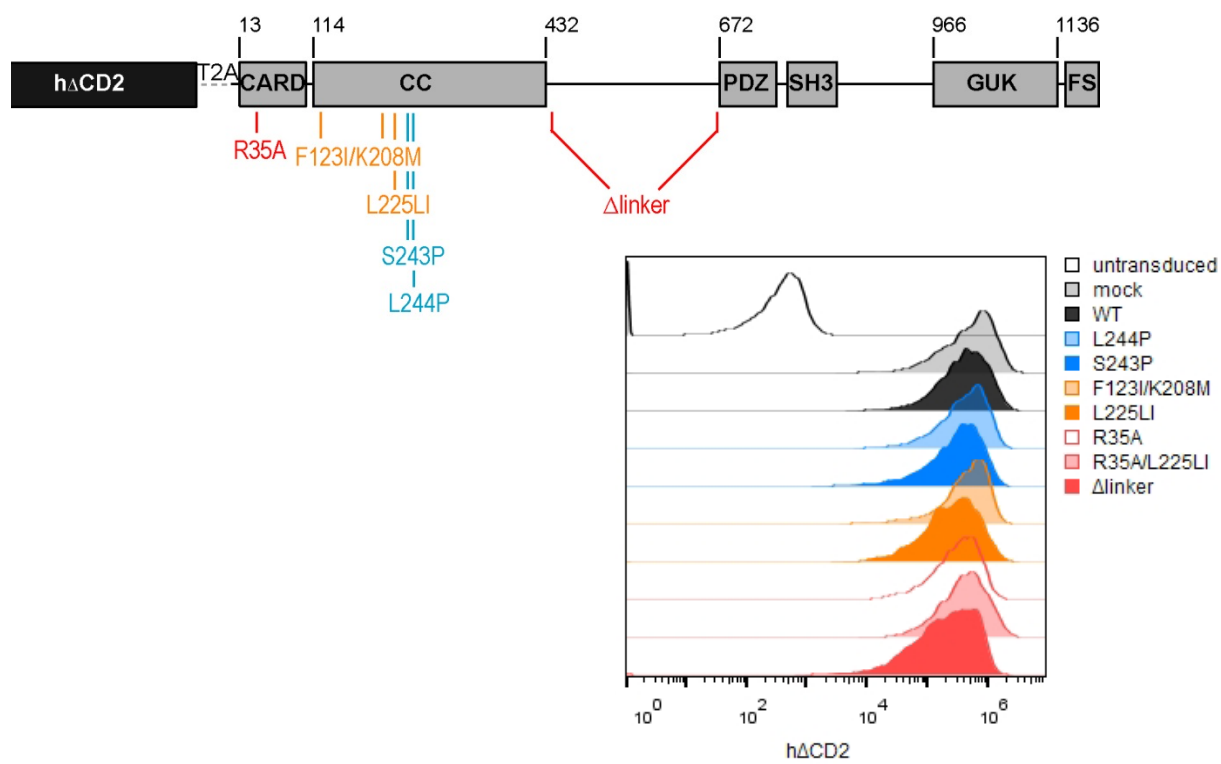

Fig. S1

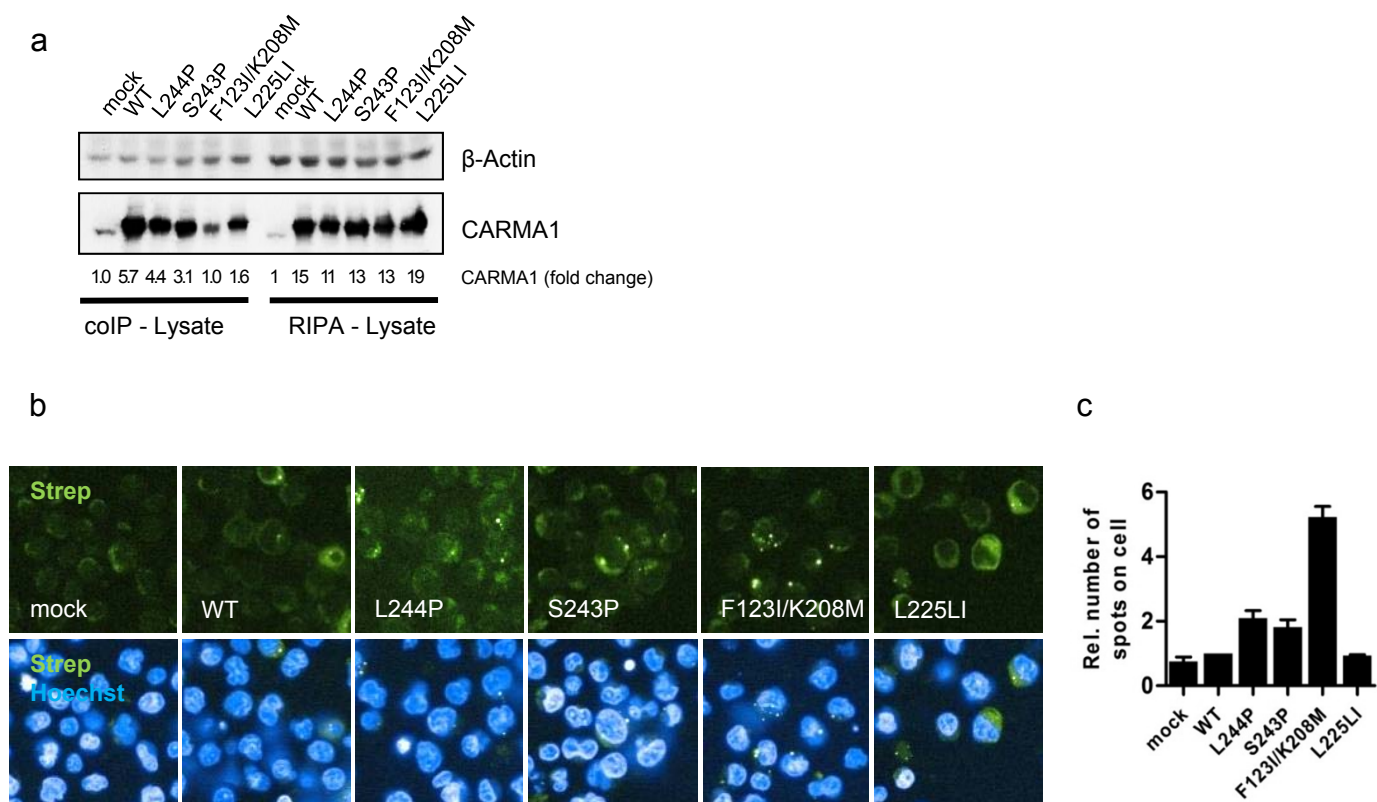

Fig. S2

a

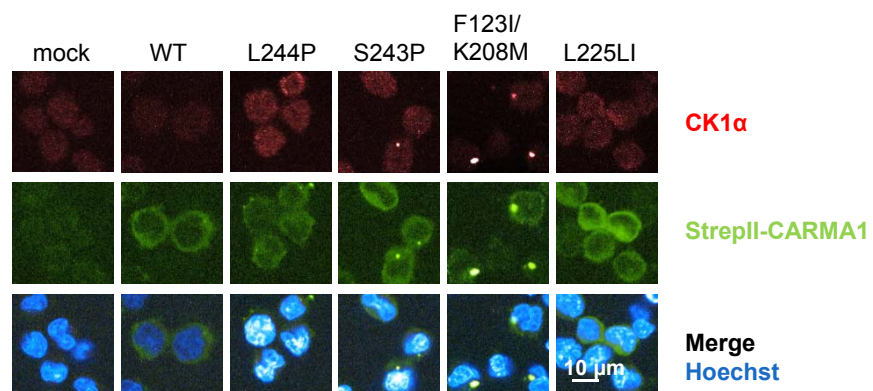

b

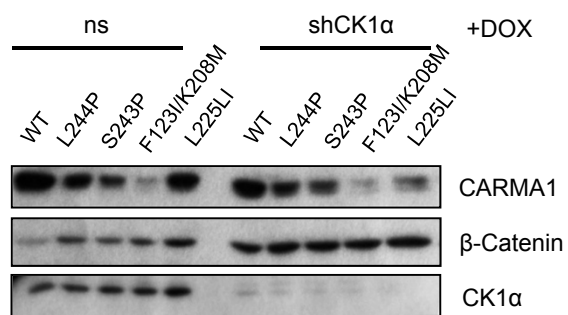

c

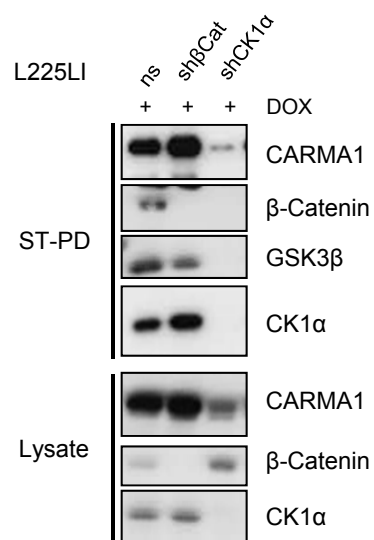

Fig. S3

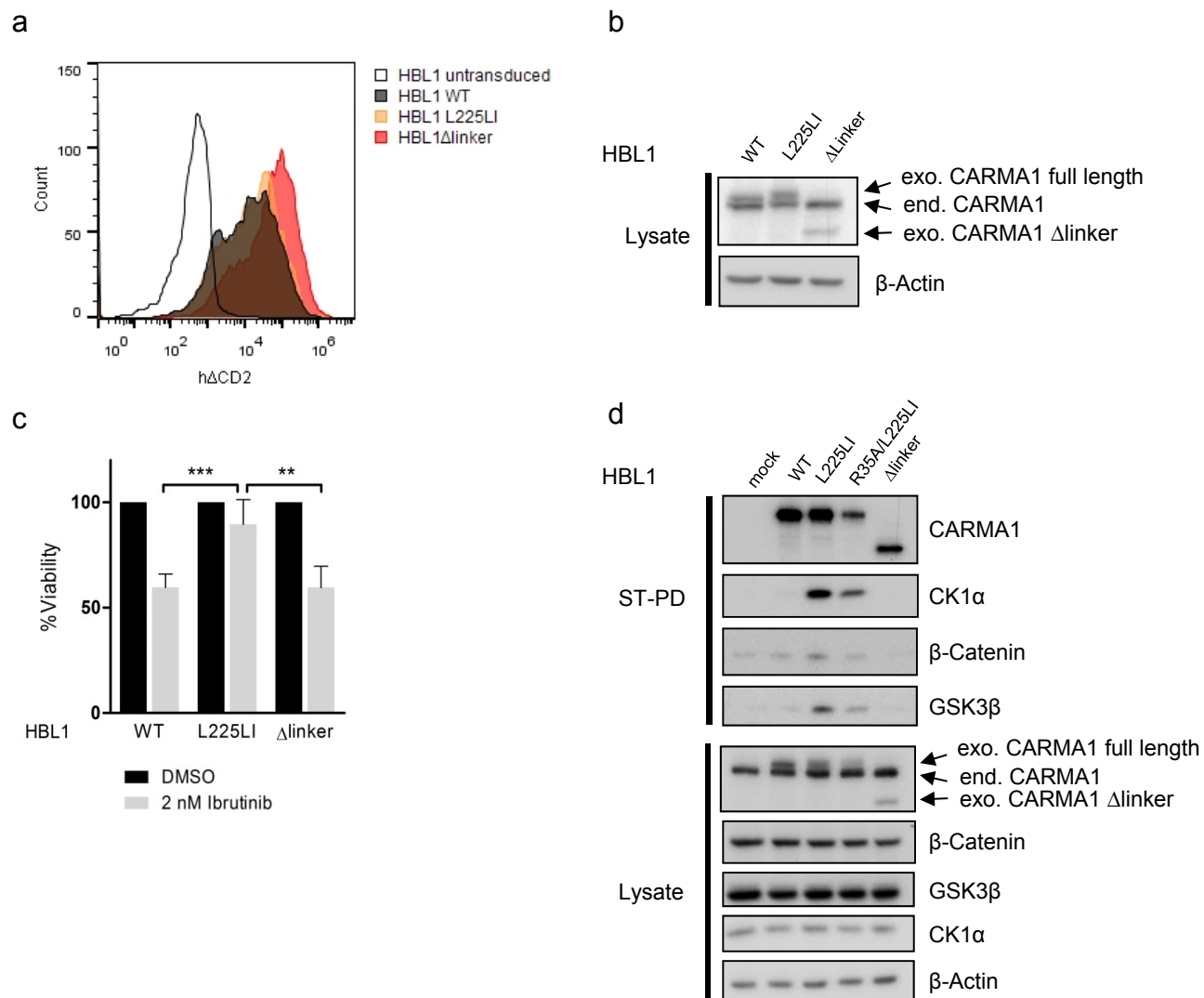

Fig. S4

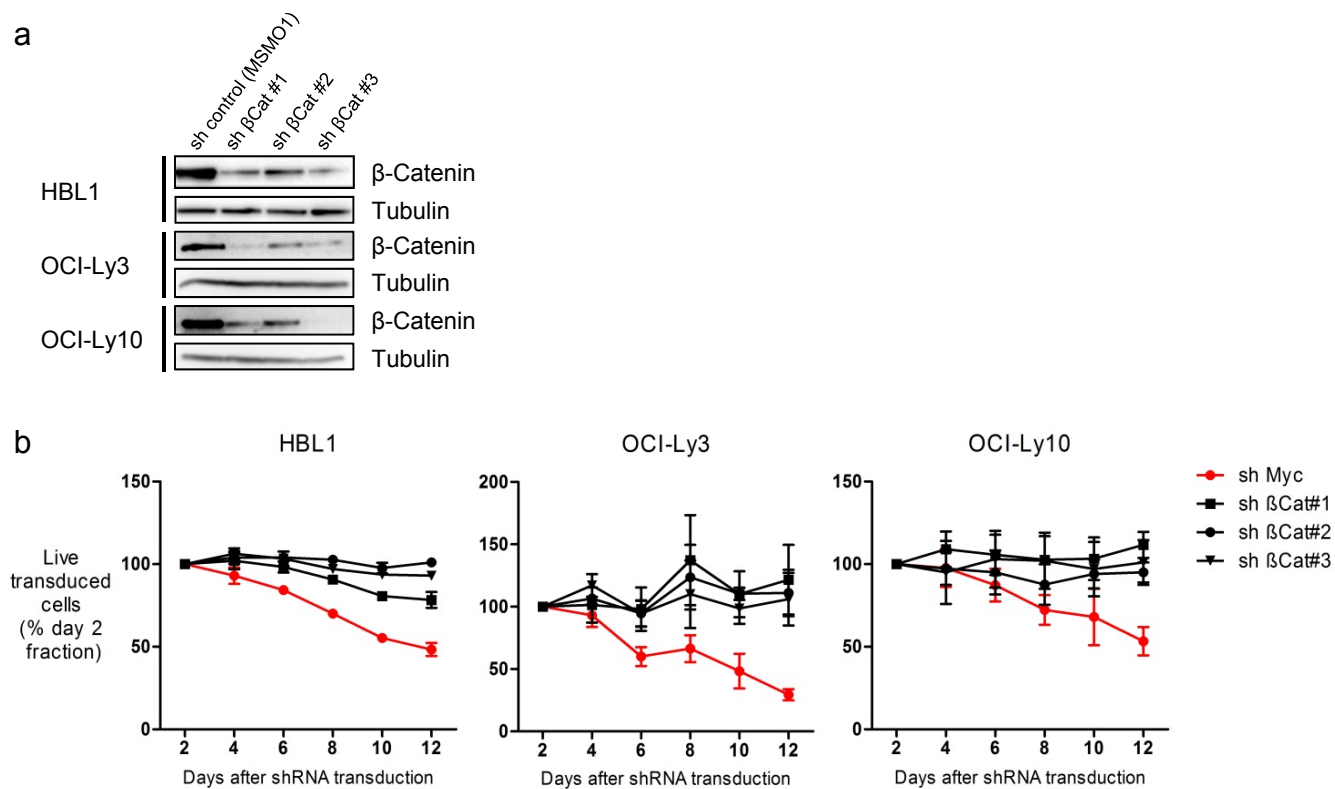

Fig. S5

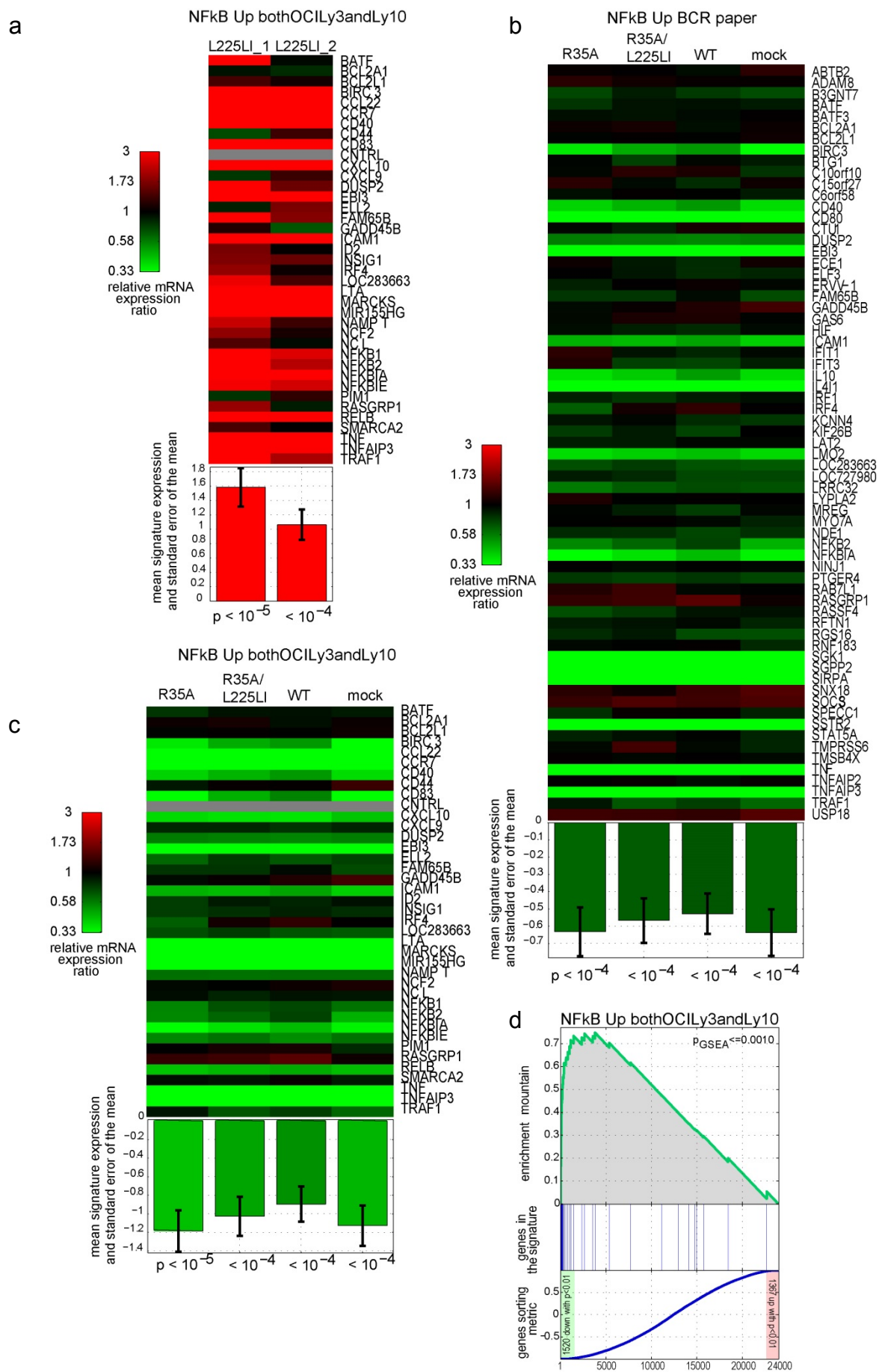

Fig. S6

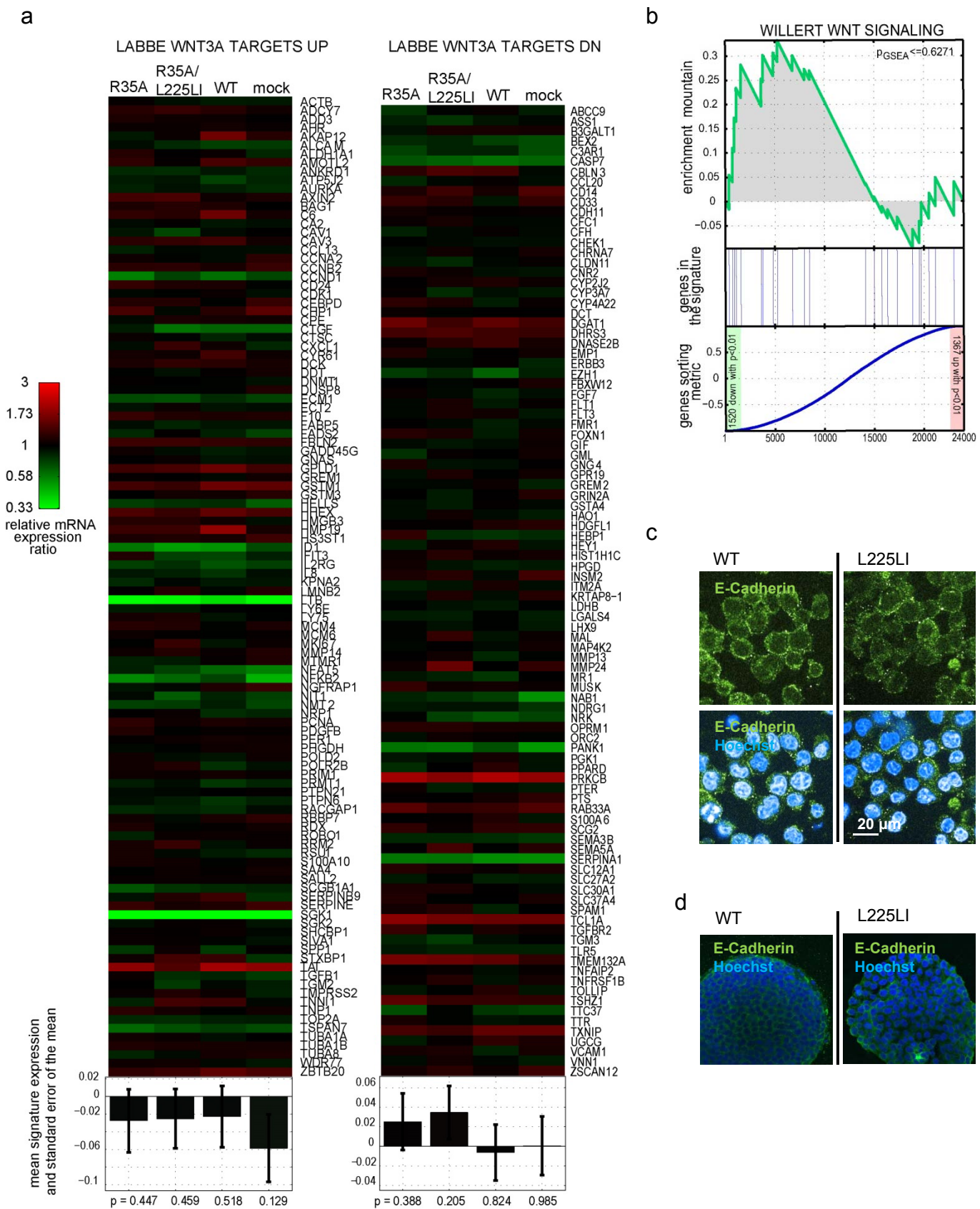

Fig. S7

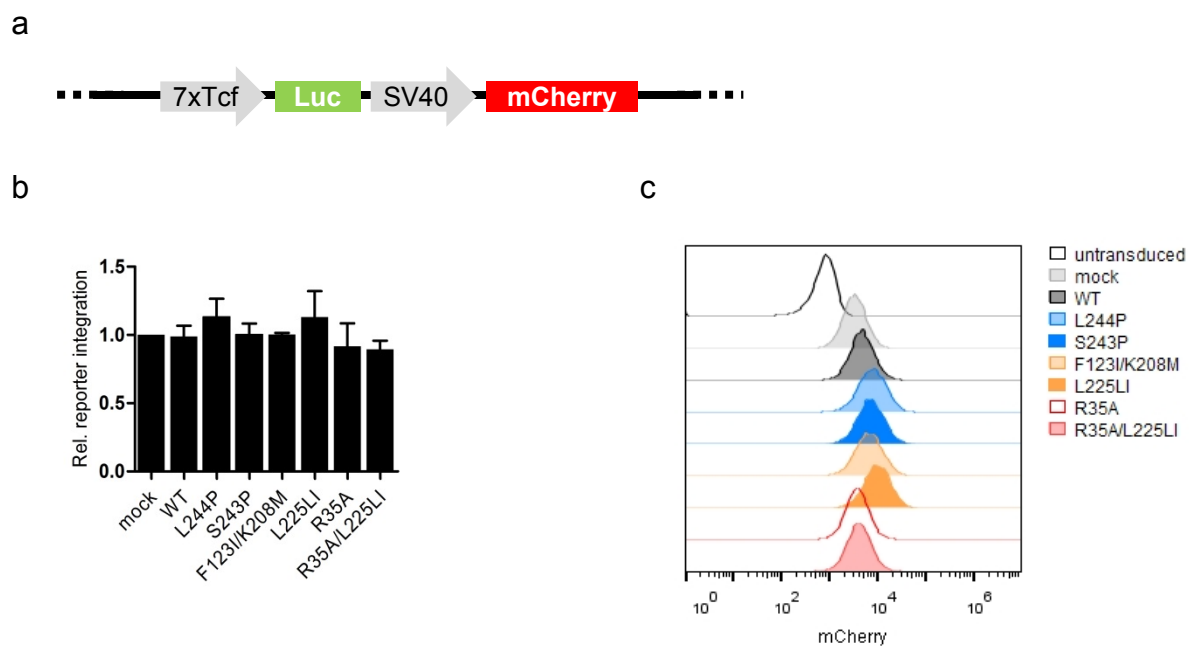

Fig. S8

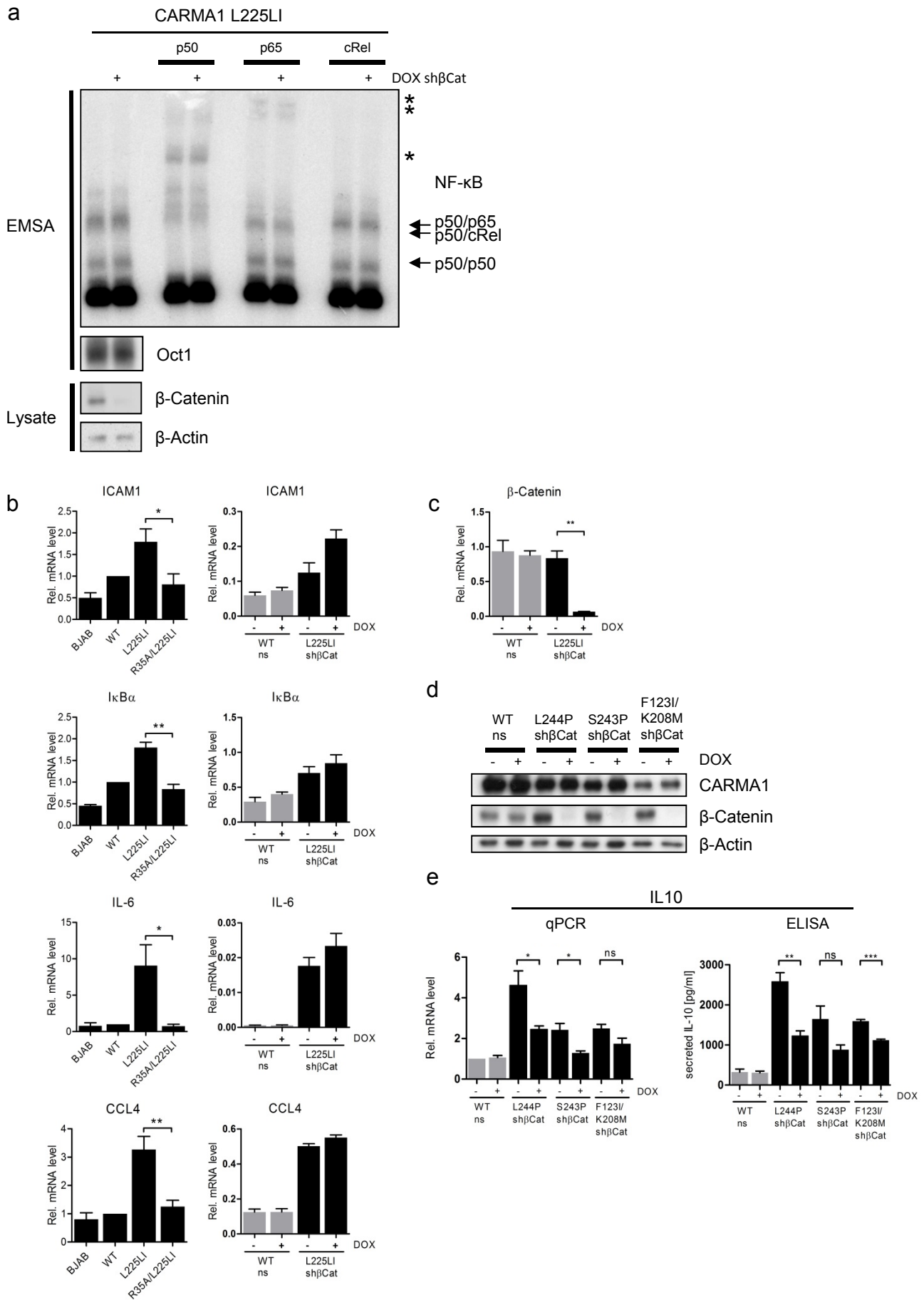

Fig. S9

Supplement: Supplementary Figures [file onc2015493x2.pdf]
